# Polypeptide templating for designer hierarchical materials

**DOI:** 10.1038/s41467-019-14257-0

**Published:** 2020-01-17

**Authors:** Hui Sun, Benedetto Marelli

**Affiliations:** Laboratory for Advanced Biopolymers, Department of Civil and Environmental Engineering, Massachusetts Institute of Technology, 77 Massachusetts Avenue, Cambridge, MA 02139 USA

**Keywords:** Biomaterials - proteins, Synthesis and processing, Polymers

## Abstract

Despite advances in directing the assembly of biomacromolecules into well-defined nanostructures, leveraging pathway complexity of molecular disorder to order transition while bridging materials fabrication from nano- to macroscale remains a challenge. Here, we present templated crystallization of structural proteins to nanofabricate hierarchically structured materials up to centimeter scale, using silk fibroin as an example. The process involves the use of ordered peptide supramolecular assemblies as templates to direct the folding and assembly of silk fibroin into nanofibrillar structures. Silk polymorphs can be engineered by varying the peptide seeds used. Modulation of the relative concentration between silk fibroin and peptide seeds, silk fibroin molecular weight and pH allows control over nanofibrils morphologies and mechanical properties. Finally, facile integration of the bottom-up templated crystallization with emerging top-down techniques enables the generation of macroscopic nanostructured materials with potential applications in information storage/encryption, surface functionalization, and printable three-dimensional constructs of customized architecture and controlled anisotropy.

## Abstract (web summary)

Despite recent progress in directed assembly of protein molecules into well-defined nanostructures, bridging materials fabrication from nano- to macroscale remains a challenge. Here, using silk fibroin as an example, the authors demonstrate templated crystallization to drive hierarchical materials growth from disordered molecules all the way up to centimeter scale.

## Introduction

Through millions of years, the natural world has developed unique methods to direct the disorder to order transition of biomacromolecules, which then allowed generation of complex materials with a variety of structural hierarchy and functionality^1^. Examples of such materials include wood, bone, tendon, silk, and mussel threads, which have constantly been a source of inspiration for the fabrication of synthetic materials that mimic the structural organization of their natural counterparts to integrate, for example, augmented mechanical properties^2^, selective mass transport^3^, energy storage^4^, and stimuli-responsiveness^5^. In natural systems, hierarchical assembly of nanoscale building blocks is at the core of materials generation and often involves the use of templates to direct the assembly pathway^6^. The same principle underpins amyloid fibrils formation through pathogen (such as prions and misfolded Aβ oligomers) templating, which follows a nucleation-elongation model^7,8^. Beyond the fundamental studies on amyloidogenesis for treatment of various neurodegenerative diseases^9^, the field of functional amyloid materials^10^ has also expanded, with interesting applications in underwater adhesives^11^, enzyme sensors^12^, and water purification membranes^13^. Inspired by protein fibrils formation with kinetic control enabled by proper templates, many novel biomimetic supramolecular assembly pathways were also developed for synthetic polymers, resulting in polymers of extremely narrow polydispersity^14,15^ and complex architectures with distinct functional domains and electronic properties^16–18^.

Despite recent progress in directed molecular assembly into well-defined nanostructures^6^, bridging materials fabrication from nano- to macroscale through a precise regulation over the disorder to order transition of biomacromolecues remains an open challenge. This is particularly true for protein-based materials, where lack of sufficient understanding of their sophisticated interactions at the molecular level makes controlled bottom–up assembly quite demanding. In addition, the delicate nature of proteins limits the use of harsh reagents and conditions commonly involved in traditional top–down nanofabrication techniques. Nonetheless, recent reinvention of structural proteins (e.g., silk fibroin) as technical materials^19^ have generated some exciting progress, although the materials achieved so far still have nontrivial limitations in either the control of protein folding and assembly at the molecular level^20,21^ or the scale-up of materials fabrication to macroscale^22^. Here, we report a template-based bottom–up approach to guide hierarchical materials growth from disordered molecules all the way up to macroscale, a process hereby termed templated crystallization. Using silk fibroin as an example, templated crystallization refers to the use of ordered peptide supramolecular assemblies to drive a phase transformation of silk fibroin from unordered to ordered conformations, thereby enabling further assembly of the silk fibroin chains into nanostructured materials (i.e., β-sheeted nanofibrils). Silk polymorphs can be engineered through templated crystallization on either homologous or heterologous peptide seeds of distinct molecular structures. Multiple parameters including the relative concentration between silk fibroin and peptide seeds, silk fibroin molecular weight (MW), and pH are investigated to fine tune the assembly kinetics and nanomechanical properties. Finally, facile integration of the templated crystallization process with emerging top–down approaches enables nanofabrication of centimeter scale nanostructured functional materials such as (i) a free-standing patterned silk film with different silk polymorphs embedded at predesigned areas, which can be used to store or encrypt information; (ii) surfaces functionalized with mesoporous and nanofibrillar silk fibroin networks obtained by epitaxial-like growth of silk fibroin on pre-deposited peptide seeds; and (iii) three-dimensional printable silk constructs with customized architecture at the macroscale and controlled anisotropy at the microscale.

## Results

### Templated crystallization of silk fibroin

**Fig. 1:**
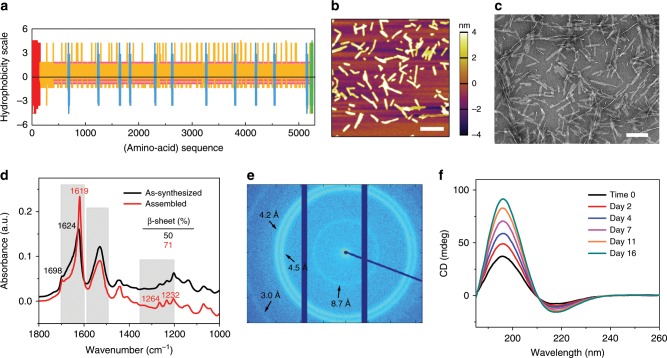
Supramolecular assembly of dodecapeptide (GAGSGA)_2_. **a** Hydrophobicity index of *Bombyx mori* silk fibroin heavy chain, which is composed of a non-repetitive (NR) N-terminal domain (red), 12 highly repetitive (HR) hydrophobic domains (orange) separated by eleven NR short hydrophilic domains (blue) in the middle, and a NR C-terminal domain (green). The dodecapeptide of sequence (GAGSGA)_2_ composes ~ 40% of the silk fibroin heavy chain sequence and the repeats were highlighted in pink, indicating its quintessential role in silk fibroin primary structure. **b** Representative atomic force micrograph of (GAGSGA)_2_ self-assembled in water, showing formation of regular nanowhisker-like supramolecular oligomers. Scale bar, 400 nm. **c** Representative negative-stain transmission electron micrograph of the (GAGSGA)_2_ nanowhiskers. Scale bar, 200 nm. **d** ATR-FTIR spectra of (GAGSGA)_2_ powders as synthesized and upon assembly from an aqueous suspension. A sharp peak centered at 1619 cm^−1^ and a strong shoulder at 1698 cm^−1^ in the Amide I band indicate a highly ordered β-sheet conformation. **e** 2-D WAXS pattern of the (GAGSGA)_2_ nanowhiskers, depicting multiple characteristic d-spacings associated with inter-strand and inter-sheet distances. **f** CD spectra of (GAGSGA)_2_ over time, revealing a typical β-sheet structure of the assembled nanowhiskers.

A proper peptide to be used for templated crystallization of silk fibroin should, in principle, (1) forms ordered supramolecular assemblies, (2) binds to silk fibroin molecules, typically through the formation of hydrogen bonds or hydrophobic interactions, and (3) shares similar isoelectric point and pH stability with silk fibroin. In this parameter space, the highly repetitive (GAGAGS)_n_ sequence of the hydrophobic domains of silk fibroin heavy chain (Fig. 1a) provides an opportunity to use peptides homologous to silk fibroin as template materials. After an initial screening (Supplementary Fig. 1), a dodecapeptide of sequence GAGSGAGAGSGA^23^ (noted as (GAGSGA)_2_ thereafter for brevity) was chosen owing to its ability to self-assemble into regular nanowhisker-like supramolecular oligomers of dimensions around 200 × 20 × 4 nm (Fig. 1b, c). Structural characterization of the nanowhiskers indicate a highly ordered β-sheet conformation, with the attenuated total reflectance Fourier transform infrared spectroscopy (ATR-FTIR) spectra depicting a sharp peak centered at 1619 cm^−1^ and a strong shoulder at 1698 cm^−1^ in the Amide I band^24^ (Fig. 1d), and the wide-angle X-ray scattering (WAXS) pattern showing multiple crystalline peaks corresponding to the inter-strand (4.2 and 4.5 Å) and inter-sheet (8.7 Å) distances^25^ (Fig. 1e). Circular dichroism (CD) spectra of (GAGSGA)_2_ in water (Fig. 1f) show a positive band centered at 196 nm and a negative band centered at 218 nm, indicating a typical β-pleated sheet structure^26^. Besides, the peptide undergoes continuous self-assembly until reaching an equilibrium, as indicated by the increase of CD signal over time. Size measurements by dynamic light scattering (DLS) show maintenance of the oligomerization state and minimal size growth of the nanowhiskers over time (Supplementary Fig. 2). Further verification of the stability of (GAGSGA)_2_ assemblies was carried out by molecular dynamics simulation^11,27^ (Supplementary Fig. 3), where system dynamics investigated by analyzing the free energy landscape of a matrix of 6 × 3 (GAGSGA)_2_ indicates that the energy was minimized in a β-sheet configuration similar to that of the hydrophobic domains of silk fibroin.

**Fig. 2:**
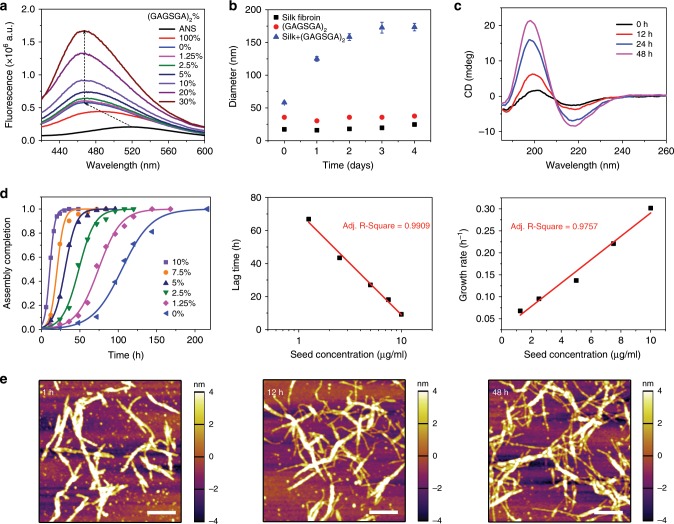
Templated crystallization of silk fibroin on (GAGSGA)_2_ nanowhiskers. **a** Fluorescence emission spectra of 8-anilinonaphthalene-1-sulfonic acid (ANS) bound to silk fibroin titrated with (GAGSGA)_2_ nanowhiskers. The increase in the fluorescence emission of ANS indicates a positive correlation between (GAGSGA)_2_ concentration and silk fibroin assembly driven by hydrophobic interactions. **b** Effective hydrodynamic diameters of silk fibroin, (GAGSGA)_2_ nanowhiskers, and silk fibroin seeded by (GAGSGA)_2_ nanowhiskers at 10%, measured by DLS over time. Error bars represent standard deviation. **c** CD spectra of silk fibroin seeded by 7.5% (GAGSGA)_2_ nanowhiskers, showing an increase in the β-sheet content of silk fibroin over time. **d** Evaluation of the kinetics of templated crystallization: fraction completion of silk fibroin assembly as a function of time at different (GAGSGA)_2_ concentrations (left), the data points were calculated from time-series CD spectra and fitted with a logistic function (solid lines); Lag time and growth rate extracted from the model fitting and plotted against (GAGSGA)_2_ concentration (middle and right, respectively). **e** Atomic force micrographs depicting a directed assembly of silk fibroin on the (GAGSGA)_2_ nanowhiskers. Thicker and longer (GAGSGA)_2_-silk nanocomplexes formed shortly after seeding (left), followed by growth of thinner silk nanofibrils branching out from the thicker (GAGSGA)_2_-silk nanocomplexes (middle and left). Scale bars, 400 nm.

The (GAGSGA)_2_ nanowhiskers with a well-defined β-sheet structure and an amino-acid sequence that composes > 40% of the silk fibroin heavy chain (Fig. 1a) were found to act as seeds that template the folding of silk fibroin in β-strands and direct material growth to form β-sheeted nanofibrils. To investigate this process, we first studied the hydrophobic-driven folding and assembly of silk fibroin exposed to different relative concentrations of (GAGSGA)_2_ nanowhiskers using 8-anilinonaphthalene-1-sulfonic acid (ANS), a fluorescent probe that undergoes an emission blue shift when it binds to hydrophobic surfaces. As depicted in Fig. 2a, the fluorescent emission peak of ANS shifted from 520 nm (ANS free in solution) to 470 nm when ANS was bound to silk^28^, and the ANS emission intensity increased when silk fibroin was exposed to nanowhiskers. This phenomenon correlates positively with the relative concentration of nanowhiskers and indicates an enhanced exposure of silk’s hydrophobic surfaces, which leads to β-sheet formation and protein assembly driven by hydrophobic interactions. Size measurements by DLS corroborated the nanowhisker-driven molecular assembly process (Fig. 2b): compared with the minimal growth of both (GAGSGA)_2_ nanowhiskers and silk fibroin micelles over time, exposing silk fibroin to 10% (weight percentage of peptide to silk fibroin) (GAGSGA)_2_ nanowhiskers caused a fast and dramatic increase in the measured hydrodynamic radius, reaching a plateau at around day 2.

The templated folding and assembly process was also characterized by time-series CD spectra, as representatively presented in Fig. 2c, where silk fibroin was exposed to 7.5% (GAGSGA)_2_ nanowhiskers, depicting a continuous increase in the β-sheet content of silk fibroin until reaching an equilibrium. CD spectra were then used to evaluate the kinetics of protein assembly^29^. Fraction completion of silk fibroin assembly at any given time and nanowhisker concentration (Fig. 2d left) was estimated assuming a two-state model^29^ (see Methods for more details), plotted and fitted with an empirical logistic function^30^

1 $$y = 1/\left( {1 + {\mathrm{exp}}\left( { - k\left( {t - t_{0.5}} \right)} \right)} \right)$$

where *k* is the growth rate, *t*_0.5_ is the time at 50% protein assembly and the lag time is calculated as

2 $$t_{\mathrm{lag}} = t_{0.5} - 1/\left( {2k} \right)$$

The lag time and growth rate extracted from the model fitting were plotted against nanowhisker concentration. Compared with the linear increase of the growth rate with nanowhisker concentration (Fig. 2d right), the lag time is found to decrease linearly with the logarithm of the nanowhisker concentration (Fig. 2d middle), similar to the aggregation behavior of the amyloid beta peptide Aβ42 within a certain range of seed concentrations^31^, indicating possible occurrence of molecular events during the lag phase of silk fibroin assembly that resemble the primary nucleation and surface-catalyzed secondary nucleation in the Aβ42 aggregation. Other models such as a variation of the Johnson-Mehl-Avrami-Kolmogorov equation^29^ can also well describe the kinetics of templated silk fibroin assembly (Supplementary Fig. 4), through which the dimensionality of growth was found to be 2 for the best fitting, indicating that the templated silk nanofibrils growth is two-dimensional in both length and width directions instead of a simple one-dimensional elongation. This was confirmed by morphological characterization of silk fibroin exposed to (GAGSGA)_2_ nanowhiskers (Fig. 2e), where silk fibroin molecules assembled on the (GAGSGA)_2_ templates and quickly led to formation of thicker and longer nanocomplexes in the beginning, followed by slower growth of thinner silk nanofibrils branching out from the thicker nanocomplexes.

### Mechanism of templated crystallization

In most of the seeded protein assembly systems, the kinetics (i.e., nucleation and growth rates) usually correlates positively with monomer concentration^7^, because if the protein assembly process is considered as a chemical reaction from monomers to polymers, the reaction rate is directly proportional to the reactant concentration. In the templated crystallization of silk fibroin, however, the assembly kinetics is found to depend negatively on monomer (i.e., silk fibroin) concentration with the same relative amount of (GAGSGA)_2_ nanowhiskers added. As shown in the CD spectra and corresponding atomic force micrographs in Supplementary Fig. 5, a random coil to β-sheet transition of silk fibroin (seeded by 5% (GAGSGA)_2_ nanowhiskers) occurred only at 0.1 and 1 mg/ml (Supplementary Fig. 5a–b), but not at 2, 5, and 10 mg/ml (Supplementary Fig. 5c-e) within the same time frame. The degree of templated silk nanofibrillization also decreases with increasing silk fibroin concentration (Supplementary Fig. 5a-e), indicating that templated assembly becomes less efficient as silk fibroin concentration increases. Nonetheless, the final concentration of silk materials obtained through templated crystallization can be increased post-nanofibrils formation by standard dehydration techniques such as ultrafiltration or simple water evaporation, as shown in Supplementary Fig. 5f, where silk nanofibrils of a β-sheet conformation are observed at a concentration of 10 mg/ml after being concentrated from 1 mg/ml.

**Fig. 3:**
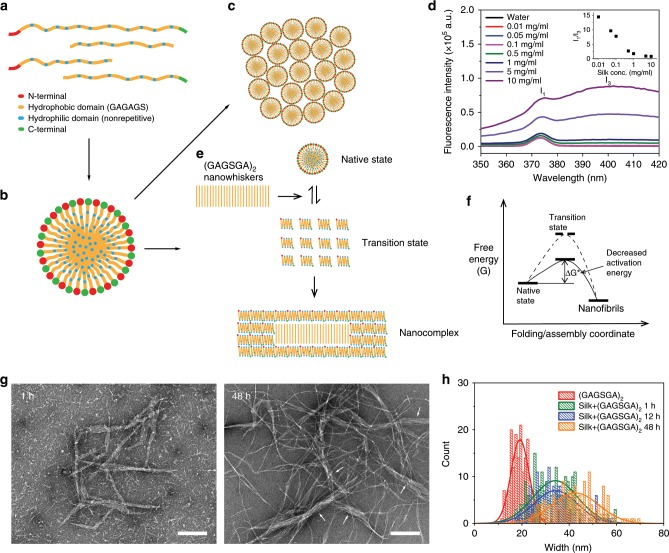
Mechanism of templated crystallization. **a** Schematic of an intact *Bombyx mori* silk fibroin heavy chain and randomly chopped chain fragments resulting from protein degradation during the regeneration process. Both native and regenerated silk fibroin were used for templated crystallization study. **b** Self-assembly of silk fibroin into micelles in water as a result of the hydrophilic–hydrophobic multi-block copolymer primary structure. **c** Micelle globule formation driven by increased silk fibroin concentration. **d** Fluorescence emission spectra of pyrene exposed to silk fibroin of increasing concentrations, from which the intensity ratio of the first (I_1_) and third (I_3_) pyrene emission peaks was calculated and plotted against silk fibroin concentration (inset). **e** Exposure to (GAGSGA)_2_ nanowhiskers decreases the activation energy for the disassembly of silk fibroin micelles (native state). Once the micelles disassemble, the extended silk fibroin chains fold into a more compact transition state (which is a β-sheet dominated state according to the CD data) to minimize the hydrophobic surfaces exposed to water. The folded silk fibroin chains are then able to build up on the (GAGSGA)_2_ nanowhiskers to form nanocomplexes. As silk fibroin concentration increases, micelles become more aggregated in the native state, therefore increasing the activation energy between the native and transition state, making random coils to β-sheet transition less favorable even in the presence of (GAGSGA)_2_ nanowhiskers. **f** Schematic free energy diagram of the templated crystallization process. **g** Negative-stain TEM images of silk nanofibrils seeded by (GAGSGA)_2_ nanowhiskers at 1 h and 48 h (left and right, respectively), for widths and lengths measurement and comparison with silk nanofibrils formed in the absence of templates (Supplementary Fig. 6). Scale bars, 200 nm. **h** Histograms of widths of (GAGSGA)_2_ nanowhiskers and (GAGSGA)_2_-silk nanocomplexes at different assembly time points.

Based on the characterization results presented in Fig. 2 and the counterintuitive dependence of the templated assembly kinetics on silk fibroin concentration, we interpret the templated crystallization process as follows (Fig. 3): above critical micelle concentration (c.m.c.), silk fibroin molecules self-assemble into micelles to minimize hydrophobic interactions with water^21,32^ (Fig. 3a–c). Exposure to highly ordered β-sheet nanowhiskers results in disassembly of silk fibroin micelles, enabling the formation of a transition state at a higher energy, where their hydrophobic domains are exposed to water before folding into a highly ordered (i.e., lower energy) β-sheet structure (Fig. 3e, f). This interpretation is supported by the emission spectra of ANS bound to silk fibroin in the presence of (GAGSGA)_2_ nanowhiskers (Fig. 2a). Assembly of folded silk fibroin chains on the nanowhisker surfaces results in thicker and longer (GAGSGA)_2_-silk nanocomplexes that are stabilized mainly by hydrophobic interactions. This process is inhibited by the formation of aggregated micelles in silk fibroin suspensions of higher concentrations^21,32^ (Fig. 3c), which causes a higher energy barrier for individual micelle disassembly. To test this hypothesis, we analyzed the fluorescence emission spectra of pyrene exposed to silk fibroin of increasing concentrations (Fig. 3d). The intensity ratio of the first (I_1_) and third (I_3_) pyrene emission peaks (intensity averaged over 371–376 nm for I_1_ and over 398–403 nm for I_3_) has been used in silk proteins to evaluate the formation of micelle-like structures^33^. Here, we found that I_1_/I_3_ decreases linearly with the logarithm of silk fibroin concentration until reaching an inflection point at ~1 mg/ml where the slope of the linear relationship changes significantly (Fig. 3d inset). For silk fibroin concentrations above this inflection point, templated crystallization and nanofibrillization become less efficient, as reported in Supplementary Fig. 5.

A comparison between silk nanofibrils templated by (GAGSGA)_2_ nanowhiskers (Fig. 3g) and those obtained by natural aging in the absence of templates (Supplementary Fig. 6) corroborated our proposed mechanism. As shown in Fig. 3g, silk fibroin molecules are able to grow on the surface of (GAGSGA)_2_ nanowhiskers and form thicker nanocomplexes as compared with the naturally aged silk nanofibrils, which have a uniform width of 7–10 nm (Supplementary Fig. 6). Widths of the (GAGSGA)_2_ nanowhiskers and (GAGSGA)_2_-silk nanocomplexes at different assembly stages were measured from the TEM images and summarized in Fig. 3h, showing a drastic thickening of the (GAGSGA)_2_ nanowhiskers by adsorption of silk fibroin in the first hour of the assembly process (from 19 ± 4 nm to 34 ± 9 nm, defined as mean ± standard deviation obtained from normal distribution fitting of ~160 data points). The quickly formed (GAGSGA)_2_-silk nanocomplexes (typically of 500–800 nm in length, Fig. 3g) are also longer than the (GAGSGA)_2_ nanowhiskers (typically of 100–300 nm in length, Fig. 1c). Assembly events at later stages are mainly growth of much thinner (7–10 nm) and longer (beyond microns) silk nanofibrils branching out from the previously formed thicker parts, whereas thickening and elongation of the (GAGSGA)_2_-silk nanocomplexes are less prominent.

### Tuning the assembly kinetics

Besides peptide nanowhisker (i.e., seed) concentration (Fig. 2d) and silk fibroin (i.e., monomer) concentration (Supplementary Fig. 5), another important parameter that can be used to actively regulate the kinetics of templated crystallization is silk fibroin MW. Here, silk fibroin regenerated from *Bombyx mori* cocoons for 30, 45, and 60 mins (named silk 30 mb, 45 mb, and 60 mb, respectively)^34^, along with native silk fibroin extracted from the gland of *B. mori* silkworms at the 5th instar were used in templated crystallization. The MW distributions of the four silk fibroins were measured by sodium dodecyl sulfate-polyacrylamide gel electrophoresis (SDS–PAGE), as shown in Supplementary Fig. 7, where the native silk fibroin shows a single band at ~390 kDa corresponding to an intact silk fibroin heavy chain, whereas the lanes of all regenerated silk fibroins show a smear of proteins having a wide MW range (Supplementary Fig. 7a). Pixel intensity analysis was performed on the gel image^35^, from which the MW distributions of all three regenerated silk fibroins were quantitatively determined (Supplementary Fig. 7b), and the MW range, average MW, and polydispersity index were calculated and summarized in Supplementary Table 1. The data show that longer cocoons degumming time corresponds to broader silk fibroin MW range, higher polydispersity, and lower average MW. Despite the differences in MW, silk fibroins regenerated with different cocoons degumming time have the same conformation, as characterized by CD (Supplementary Fig. 8). The CD spectrum of native silk fibroin is slightly different from that of regenerated silk fibroin, but still shows an unordered conformation. To investigate the effects of silk fibroin chain length and polydispersity on the templated crystallization process, the four silk fibroins were seeded by the same amount of (GAGSGA)_2_ nanowhiskers and the folding and assembly processes thereafter were characterized by CD, from which the kinetics profiles were determined (Supplementary Fig. 9a). By fitting the kinetics data with the same model used earlier, lag time and growth rate were obtained and plotted in Supplementary Fig. 9b. Compared with regenerated silk fibroin, nucleation in native silk fibroin barely has lag time and nanofibrils growth thereafter is also faster (14 times faster than silk 60 mb). Among the regenerated silk fibroins, smaller and more-degraded silk fibroin corresponds to longer lag time and slower growth rate (i.e., slower templated crystallization rate).

Other factors that affect the kinetics of templated crystallization and nanofibrillization include milieu pH, where templated assembly of silk fibroin is found to be hindered in basic environments and the polymorphic phase transformation of silk fibroin becomes slower as the milieu pH increases (Supplementary Fig. 10a). Besides, increased pH restricts nanofibrils growth in length and mitigates the overall aggregation and association of mature silk nanofibrils (Supplementary Fig. 10b and c). In addition, we note that heating the (GAGSGA)_2_ solution at 80 ˚C for 20 mins results in disassembly of the nanowhiskers^23^, yielding separated and unstructured peptides (Supplementary Fig. 11a) that do not act as seeds and initiate templated crystallization of silk fibroin (Supplementary Fig. 11b).

### Engineering silk polymorphs

**Fig. 4:**
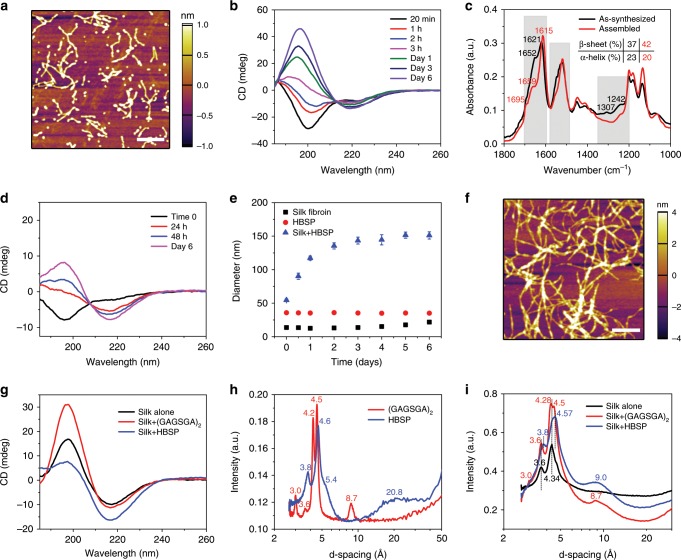
Structural characterization of HBSP and silk fibroin seeded by HBSP. **a** Representative atomic force micrograph of HBSP self-assembled in water, showing that the peptide self-assembles into oligomers of irregular shapes and dimensions. Scale bar, 400 nm. **b** CD spectra of HBSP depicting its structural evolution. HBSP is mostly unordered right after dissolution in water and then assembles into a β-sheet dominant structure through intermediate α-helical dominant states (e.g., at 2 h). **c** ATR-FTIR spectra of HBSP powders as synthesized and upon assembly from an aqueous suspension, showing a combination of β-sheet (main peaks at 1621 and 1615 cm^−1^) and α-helix (shoulders at 1652 and 1659 cm^−1^) conformations. **d** CD spectra of silk fibroin seeded by 10% HBSP, depicting an increase in the molecular order over time, but with a lower β-sheet content compared to silk fibroin seeded by (GAGSGA)_2_. **e** Effective hydrodynamic diameters of silk fibroin, HBSP nanoassemblies, and silk fibroin seeded by 10% HBSP, measured by DLS over time. Error bars represent standard deviation. **f** Representative atomic force micrograph of silk fibroin seeded by HBSP nanoassemblies, showing mature nanofibrils at 48 h. Scale bar, 400 nm. **g** CD spectra of naturally aged silk fibroin in the absence of templates, (GAGSGA)_2_- and HBSP-templated silk fibroin, revealing different β-sheet contents (i.e., molecular order). **h** 1-D WAXS spectra of (GAGSGA)_2_ and HBSP nanoassemblies, showing distinct intra-/intermolecular distances. **i** 1-D WAXS spectra of naturally aged silk fibroin, (GAGSGA)_2_- and HBSP-templated silk fibroin, depicting different molecular packing (i.e., d-spacings) of silk fibroin, owing to the templates’ effects.

To demonstrate the universality of templated crystallization in silk fibroin, a heterologous peptide of sequence ALKAQSEEEAASARANAATAATQSALEG was investigated. The peptide sequence originates from the silk fibroin AmelF3 of European honeybee *Apis mellifera*, which is one of the four proteins that form a coiled-coil tetramer^36–38^. For simplicity, this peptide is named honeybee silk peptide (HBSP) thereafter. Similar to (GAGSGA)_2_, HBSP is also able to undergo supramolecular self-assembly to form oligomers (Fig. 4a). The morphology of the oligomers, however, is not as regularly defined as that of (GAGSGA)_2_ nanowhiskers, with the appearance of both elongated nanorods and nanoparticles. Besides, the HBSP nanoaggregates are thinner than the (GAGSGA)_2_ counterparts in terms of width and height. The elongated HBSP oligomers also appear to be more flexible owing to the curved morphologies, as compared to the straight and thick (GAGSGA)_2_ nanowhiskers. CD spectra of HBSP solution over time (Fig. 4b) show a folding and assembly of HBSP from a mostly unordered conformation to a final β-sheet dominant structure through α-helix dominant intermediate states (e.g., 2 h). ATR-FTIR characterization of HBSP (Fig. 4c) corroborates the structural information obtained from CD, depicting a combination of β-sheet and α-helix as the major secondary structure components. Templated folding and assembly of silk fibroin on HBSP was then investigated using the same set of characterization techniques (Fig. 4d–f). Specifically, CD spectra of silk fibroin seeded by 10% HBSP show an increase in the β-sheet content of silk fibroin over time (Fig. 4d), but with much lower β-sheet content compared with silk fibroin seeded by (GAGSGA)_2_ at corresponding time points. DLS characterization of silk fibroin mixed with HBSP (Fig. 4e) show a similar trend as that observed when mixing silk fibroin with (GAGSGA)_2_. The increase in the measured hydrodynamic size is again owing to the formation of silk nanofibrils, as shown in Fig. 4f.

To investigate the structural differences of the end materials templated by (GAGSGA)_2_ and HBSP, CD spectra of silk fibroin were collected 1-month after being mixed with the two peptide seeds (Fig. 4g). The spectrum of naturally aged silk in the absence of any templates was also reported in Fig. 4g for comparison, indicating that the final structures of silk fibroin are not only affected by the addition of templates, but also by their structures. Moreover, WAXS was performed on silk nanofibrils obtained by templated crystallization on different seeds as well as natural aging to demonstrate the dependence of the molecular structure of the end materials on that of the templates (Fig. 4h, i). The results show that the intermolecular arrangements in the templated silk nanofibrils are able to follow that of the peptide seeds, as indicated by the appearance of new d-spacings in the spectrum of silk fibroin templated by (GAGSGA)_2_ and the peak shifts (3.6 Å to 3.8 Å and 4.34 Å to 4.57 Å) observed in the spectrum of silk fibroin templated by HBSP, compared with the spectrum of naturally aged silk.

### Nanomechanical characterization of silk nanofibrils

**Fig. 5:**
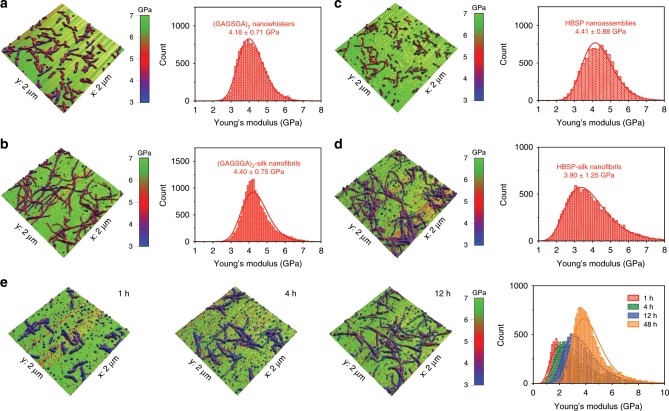
Nanomechanical characterization of the peptide seeds and associated silk nanofibrils. Young’s modulus maps overlaid on 3D topography (left) and histograms of the Young’s moduli (right) for: **a** (GAGSGA)_2_ nanowhiskers. **b** (GAGSGA)_2_-templated silk nanofibrils. **c** HBSP nanoassemblies. **d** HBSP-templated silk nanofibrils. **e** Silk nanofibrils seeded by (GAGSGA)_2_ nanowhiskers at intermediate time points. The Young’s moduli in each histogram (typically of ~ 10,000 data points) were fitted with a log-normal distribution and reported as mean ± standard deviation.

Nanomechanical properties of the peptide seeds and silk nanofibrils obtained through templated crystallization were measured by atomic force microscopy (AFM)-based bimodal tapping mode force microscopy, namely amplitude modulation-frequency modulation (AM-FM) mapping^39,40^. For the peptide seeds deposited on mica, AM-FM mapping reported Young’s moduli of 4.18 ± 0.71 GPa (defined as mean ± standard deviation obtained from log-normal distribution fitting of ~ 10,000 data points) for (GAGSGA)_2_ nanowhiskers (Fig. 5a) and 4.41 ± 0.88 GPa for HBSP nanoaggregates (Fig. 5c), indicating a similar stiffness for the two peptide oligomers in the transverse direction, although the HBSP nanoaggregates appear to be more flexible axially. Silk nanofibrils obtained by templated crystallization on the two seeds, however, have different Young’s modulus. Specifically, AM-FM mapping reported a lower modulus mean and larger modulus variance for the silk nanofibrils templated by HBSP (Fig. 5d), as compared with the silk nanofibrils templated by (GAGSGA)_2_ (Fig. 5b), corroborating the structural differences found with CD and WAXS (Fig. 4g–i) as the nanofibrils stiffness correlates positively with the β-sheet content present^41^ and is strongly affected by intermolecular arrangements. Besides, silk nanofibrils showed an increase in their stiffness over time (Fig. 5e), which is believed to be associated with the increase in β-sheet contents (i.e., higher order in the protein structure) as the templated assembly proceeds. In addition, ratios of indentation depth to nanofibril height for each AM-FM scan were calculated and found to be in the range of 0.1–0.3 (Supplementary Fig. 13), indicating that there should not be much substrate “see-through” effect^42^ on the measured moduli for the peptide seeds and silk nanofibrils. Besides, spin-coated polystyrene (PS) films that have a similar Young’s modulus to silk fibroin (Supplementary Fig. 14) were also used as substrates for AM-FM mapping (Supplementary Fig. 15), which reported similar stiffness values to those measured on mica.

Following the study of how silk fibroin chain length and polydispersity affect the templated crystallization process, mechanical properties of nanofibrils assembled from native silk fibroin and regenerated silk fibroins of different degumming time were also measured. As presented in Supplementary Fig. 12, native silk nanofibrils have higher modulus mean and smaller modulus variance than regenerated silk nanofibrils; for regenerated silk fibroin, longer degumming time results in lower modulus mean and larger modulus variance of the obtained nanofibrils, which can be explained by the more severe protein degradation and broader MW distribution induced by longer cocoons degumming. In all cases, silk nanofibrils obtained with templated crystallization possessed a slightly higher Young’s modulus compared with the ones measured for other nanofibrillar proteins and amyloids^43–45^. Nonetheless, the extremely small indentation depths (typically < 5 nm) applied by the AFM tips makes the modulus measurement by AM-FM prone to errors and variations caused by surface effects such as roughness, adhesion, and contaminations^39^, so comparison of the absolute modulus values measured by different techniques should be performed with caution.

In light of various applications that require silk fibroin-based materials to perform in hydrated state, effects of hydration on the morphology and stiffness of the peptide/silk assemblies were investigated. Specifically, cryo-EM and negative staining were used to compare the morphology and dimensions of the (GAGSGA)_2_ nanowhiskers and the templated silk nanofibrils in hydrated and dried state (Supplementary Fig. 16), from which no apparent difference was observed. Mechanical properties of the peptide/silk assemblies, however, are significantly different depending on the environment. Compared with the dried (GAGSGA)_2_ nanowhiskers and (GAGSGA)_2_-templated silk nanofibrils of GPa scale stiffness, the same materials in hydrated state become much softer with the stiffness dropping down to 1–2 MPa (Supplementary Fig. 17), which is common for proteinaceous materials. As a reference for the nanomechanical measurements on individual silk nanofibrils, which may contain certain level of inaccuracy owing to the very shallow indentation depths, stiffness of bulk gels formed by templated crystallization of silk fibroin on (GAGSGA)_2_ were also measured with AFM. Representative force-indentation curve of the silk gel was given in Supplementary Fig. 18a and analyzed with a Hertz model to get the Young’s modulus. Gaussian fitting of the Young’s moduli obtained from 216 force-indentation curves reported 5.96 ± 0.48 MPa for the silk gel (Supplementary Fig. 18b), and the use of other contact mechanics models such as Johnson-Kendall-Roberts (JKR) and Oliver-Pharr produced similar moduli – 6.26 ± 0.49 and 5.97 ± 1.92 MPa, respectively.

### Nanofabrication of macroscopic hierarchical materials

**Fig. 6:**
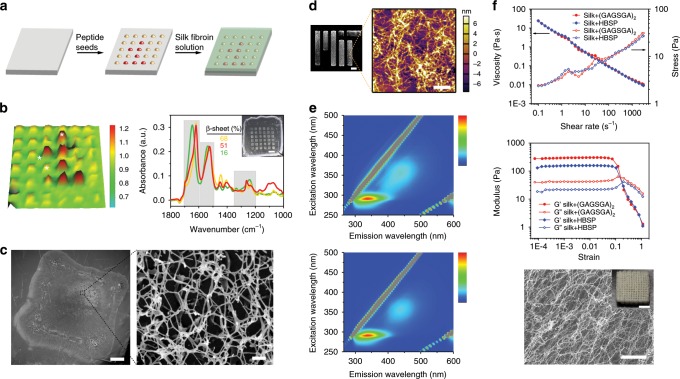
Engineering silk nanofibrils into macroscopic materials. **a** Schematic of the process for epitaxial-like growth of silk fibroin on substrates modified with peptide seeds. Yellow and red denote (GAGSGA)_2_ and HBSP seeds, respectively. **b** ATR-FTIR map of a patterned silk film (inset in the right panel, scale bar, 3 mm) fabricated by the process shown in **a** revealing the hidden Arabic numeral “1”, which is indiscernible by visual or microscopic inspection. The color scale represents the ratio of absorbance intensity at 1523 cm^−1^ to that at 1536 cm^−1^ (Amide II peaks). Representative spectra at the three asterisks in the FTIR map were given in the right panel, showing phase transformation of silk fibroin from random coils to β-sheet in the patterned areas, and (GAGSGA)_2_ seeds template silk fibroin into a higher β-sheet content compared to HBSP seeds. **c** SEM characterization of a surface functionalized with mesoporous and nanofibrillar silk fibroin that grows on the pre-deposited seeds. Scale bars, 200 μm (left) and 300 nm (right). **d** Inkjet printing of a silk nanofibrils suspension obtained through templated crystallization. Scale bars, 1 mm (left) and 1 μm (right). **e** Fluorescence excitation–emission matrix (EEM) of suspensions of silk nanofibrils seeded by (GAGSGA)_2_ (upper panel) and HBSP (lower panel), where the excitation and emission maxima at 355 and 438 nm, respectively (i.e., fluorescence in the visible range) indicate the presence of highly β-sheeted (i.e., hydrogen bond rich) protein fibrils. The color scale represents fluorescence intensity (arbitrary unit). **f** Evaluation of the rheological properties of silk nanofibrils gels: shear viscosity and stress as a function of shear rate, depicting a shear-thinning behavior (upper panel); Storage and loss moduli (G’ and G”, respectively) obtained by oscillation strain sweep at fixed frequency, as a measurement of viscoelasticity (middle panel); continuous printing of the silk nanofibrils gel into a three-dimensional construct (lower panel, inset) and a representative SEM image of the internal structure, showing an anisotropic nanofibrillar network. Scale bars, 5 mm (inset) and 200 nm (SEM).

Templated crystallization as a bottom–up molecular assembly process can be easily combined with various top–down manufacturing techniques to generate macroscopic hierarchical materials with complex shapes and predesigned nanostructures. In particular, two different strategies were pursued: controlled deposition of peptide seeds on a solid surface allows to topographically regulate the growth of silk nanofibrils and form a nanostructured protein overlayer, in a way that resembles epitaxial growth of inorganic materials. Alternatively, suspensions or gels composed of silk nanofibrils obtained by templated crystallization can be developed as printable inks and integrated with printing techniques to fabricate three-dimensional constructs with customized architecture at the macroscale and controlled anisotropy at the microscale. The results are summarized in Fig. 6, demonstrating effective employment of templated crystallization in various nanofabrication approaches.

For the epitaxial-like growth of silk fibroin on substrates modified with peptide seeds, (GAGSGA)_2_ nanowhiskers and HBSP nanoassemblies were selectively deposited on polydimethylsiloxane (PDMS) following a predesigned pattern (Fig. 6a). The peptide-grafted substrate was then exposed to silk fibroin solution, allowing for templated crystallization of silk fibroin on the underlying seeds. This process enables nanofabrication of centimeter scale patterned free-standing silk films with programmable material morphology, mesoporosity, molecular structure and crystallinity (Fig. 6b inset), which can also be used to store or encrypt information. As a proof-of-concept, HBSP seeds were deposited on PDMS to form a pattern of Arabic numeral “1” in the checkerboard background composed of (GAGSGA)_2_ seeds (Fig. 6a). The fabricated free-standing silk film (Fig. 6b, inset) was able to acquire the background pattern owing to the polymorphic phase transformation of silk fibroin from random coils to β-sheet, changing the transparency of the silk film. The embedded numeral “1”, however, was indiscernible in the patterned silk film by visual or microscopic inspection. Nonetheless, the numeral “1” becomes evident (Fig. 6b, left) with ATR-FTIR mapping of the patterned silk film, which is able to identify differences in molecular structures. The color scale of the FTIR map (Fig. 6b left) represents the ratio of absorbance intensity at 1523 cm^−1^ to that at 1536 cm^−1^ (Amide II peaks). Representative spectra at the three asterisks in the FTIR map were included in the right panel of Fig. 6b, depicting phase transformation of silk fibroin from random coils to β-sheet corresponding to the patterned areas. The β-sheet contents for (GAGSGA)_2_-patterned areas, HBSP-patterned areas, and non-patterned areas were measured to be 68, 51, and 16%, respectively (Fig. 6b, right). Depending on the peptide seeds used, the crystallinity (i.e., β-sheet content) of the final silk fibroin materials can be effectively controlled, with both cases achieving a crystallinity comparable to that of ethanol-treated silk films or silk fibers (Supplementary Fig. 19). Alternatively, the epitaxial growth process can be used to functionalize a surface with mesoporous and nanofibrillar silk fibroin (Fig. 6c) obtained by templated crystallization on the pre-deposited seeds, with potential applications in antifouling coatings, nanofiltration, and so on.

Another demonstration for scaling up nanomaterials fabrication to macroscale involves the development of printable inks that can be used to build three-dimensional nanostructured constructs with customized architecture at the macroscale and controlled anisotropy at the microscale. The process to achieve such materials encompasses (1) formation of silk fibroin nanofibrils in suspension through templated crystallization, (2) concentrating the suspension to achieve desired rheological properties, (3) use of the obtained bioink in combination with various printing techniques (4) tuning the printing parameters to achieve nanofibrils alignment at the microscale. As a first proof-of-concept, silk nanofibrils suspension was coupled with inkjet printing^46^ to deposit nanofibrillar mats with a spatial resolution of 10 microns in the *x*–*y* plane and 30 nm in the *z* direction (Fig. 6d). To enable continuous printing of silk nanofibrils for the formation of three-dimensional hierarchical materials, a gel-like ink was developed by concentrating the silk nanofibrils suspension through a simple water evaporation process, during which a sol–gel transition occurs at ~5 mg/ml and is marked by the appearance of visible macrophases (Supplementary Fig. 20). The gel-like behavior at concentrations as low as 5 mg/ml is mainly owing to the densely packed nanofibrillar networks as characterized by the fluorescence excitation–emission matrix (EEM, Fig. 6e), where the excitation and emission maxima at 355 and 438 nm, respectively (i.e., fluorescence in the visible range) are characteristic of the existence of hydrogen bond-rich protein fibrils^47^. This is also confirmed by comparison with the fluorescence EEM of amorphous silk fibroin solution in which no nanofibrils exist (Supplementary Fig. 21), where there are only excitation and emission maxima at 290 and 350 nm, respectively, owing to the presence of tyrosine and tryptophan in the protein^48^. Besides, the area and intensity of the fluorescence maxima at 355/438 nm are indicative of the amount of β-sheets (or equivalently hydrogen bonds) in the silk nanofibrils templated by different peptide seeds (upper and lower panel correspond to silk nanofibrils templated by (GAGSGA)_2_ and HBSP, respectively). Rheological studies of the nanofibrillar gels show that both gels exhibit a shear-thinning behavior with a similar viscosity of ~ 30 Pa∙s at shear rate of 0.1 s^−1^ (Fig. 6f upper panel), orders of magnitude higher than the viscosities of regenerated silk fibroin solution which shows minimal shear-thinning property (Supplementary Fig. 22). The storage modulus (G’) of both silk nanofibrils gels are about seven times higher than their loss modulus (G”) in the linear viscoelasticity region (Fig. 6f middle panel), depicting a more solid-like behavior of both gels. In addition, the G’ and G” of (GAGSGA)_2_-templated silk gels are about twice the moduli of HBSP-templated gels, indicating a dependence of the macroscopic mechanical properties on the microscopic structures. The good elasticity of the silk gels enabled continuous printing of silk nanofibrillar materials with an extrusion-based bioprinter, as shown in the inset of Fig. 6e lower panel. SEM characterization of the printed materials confirmed the deposition of nanofibrillar silk fibroin, where the nanofibrils could also be aligned by controlling the nozzle dimension and printing speed (Fig. 6e lower panel). Moreover, printed silk showed enhanced intrinsic fluorescence (Supplementary Fig. 23a) that results from the highly β-sheeted structure of the nanofibrils, as previously observed in amyloid fibrils^11^. It also displayed form birefringence^49^ (Supplementary Fig. 23b), owing to the refractive index difference between silk fibroin (*n* = 1.54)^50^ and air as a result of the mesoporous feature, along with the orientational arrangement of silk nanofibrils^20^ induced by the shear-force during the printing process.

## Discussion

Templated crystallization enables the design and fabrication of nanostructured silk fibroin materials through a precise regulation over protein disorder to order transition. Facile integration of templated crystallization with emerging top–down techniques allows the engineering of macroscopic hierarchical protein-based materials with control over molecular structures, surface morphology, mesoporosity, and mechanical properties. The capability to grow and print silk nanofibrils of different polymorphs on demand and into customized shapes is essential for engineering the next generation smart and compartmentalized devices. Nanomanufacturing with templated crystallization also promises to be extended to other natural or synthetic polymer systems where highly ordered molecular structures can be achieved, facilitating nanofabrication of multiscale and complex materials with programmable functions.

## Methods

### Peptide synthesis

All the peptides used in this study were synthesized by GenScript (Piscataway, NJ), with free N- and C termini. In brief, peptides were synthetized using standard Fluorenylmethyloxycarbonyl (Fmoc)-based solid-phase peptide synthesis and purified by reverse-phase high-performance liquid chromatography to a purity of 95% or higher. All peptides were dissolved in pure Milli-Q water.

### Silk fibroin preparation

The regenerated silk fibroin used in this study was extracted from *B. mori* cocoons following established protocols. In brief, chopped silk cocoons were degummed in a boiling 0.02 m sodium carbonate solution for 30–60 mins to remove the sericin. The obtained silk fibers were then washed with Milli-Q water for several times followed by overnight drying. The dried silk fibers were dissolved in 9.3 m lithium bromide for 4 h at 60 ˚C followed by dialysis against Milli-Q water for 2 days with constant changing of water. The resulting silk fibroin solution was then purified by sequential centrifugation at 4800 × *g* and 20,000 × *g* for 25 mins each, yielding a final silk fibroin solution of ~ 7 wt%. The regenerated silk fibroin solution was then stored at 4 ˚C until use. Unless otherwise noted in the text, the silk fibroin used in all experiments were degummed for 30 mins (30 mb).

Native silk fibroin was extracted from *B. mori* silkworms at the beginning of 5th instar. In brief, silkworms were dissected for their glands, which were then transferred to cold (4 ˚C) Milli-Q water. After 5 mins, the epithelial membrane was carefully peeled off from the gland using tweezers. The posterior and anterior parts of the glands were then separated off and discarded. The middle glands were washed in Milli-Q water for another 30 mins to remove the sericin coating, after which they were stored at 4 ˚C and used within a day.

### Gel electrophoresis

The MW distributions of native and regenerated silk fibroins were measured by SDS–PAGE using NuPAGE 3–8% Tris-Acetate Protein Gels (Invitrogen). Sample preparation followed the protocols from the gel manufacturer. HiMark Pre-stained Protein Standard (Invitrogen) was used as the ladder. The gel was run under reducing conditions for 60 mins at 150 V, stained with a Colloidal Blue staining kit (Invitrogen) and imaged by ChemiDoc (Bio-Rad). To determine the MW distributions from the gel image, pixel intensity as a function of lane position was obtained using Matlab. The reference lane containing protein standards was used to establish a conversion between pixel position and molecular weight. Pixel intensity was normalized to be in the range of 0–1 and a threshold of pixel intensity was applied, above which the pixels were considered as background.

### Molecular dynamics simulation

The initial model for the (GAGSGA)_2_ oligomer was constructed by threading the dodecapeptide sequence onto the structure of a poly-(Gly-Ala) β-sheet (Protein Data Bank identification code 2slk) using UCSF Chimera and Modeller. The system was then equilibrated in a transferable intermolecular potential 3 P (TIP3P) explicit water box. Simulations were run for 60 ns with a time-step of 2 fs at constant temperature (300 K) and pressure (1 bar) using GROMACS. The force field used was CHARMM27. The stability of the β-sheet assembly was verified from hydrogen bond dynamics and root mean squared deviation data obtained from the molecular dynamics trajectory.

### Imaging

Scanning electron microscopy (SEM) images were obtained using a Zeiss Merlin High-resolution SEM. Samples containing liquid were first quickly frozen in liquid nitrogen to minimize changes in mesoscopic structures, followed by lyophilization at − 105 ˚C and vacuum condition using a freeze drier (FreeZone, LABCONCO). All samples were sputtered coated with a thin layer of gold (5 nm) before imaging.

AFM images were obtained using a Cypher S AFM (Asylum Research). Samples were diluted to around 30 μg/ml (for silk fibroin) and 15 μg/ml (for peptide oligomers) to have the optimal feature densities on the substrate for topography imaging. A 10 μL aliquot of diluted samples was dropped on freshly cleaved mica surface (*ϕ* = 10 mm, Ted Pella) and dried before imaging. All morphological characterization were performed by tapping mode in air, at a scan rate of 2.0 Hz and a resolution of 256 × 256 pixels per image, using AC160TS-R3 (Olympus) probes.

Negatively stained transmission electron microscopy (TEM) images were recorded using a Tecnai G^2^ Spirit TWIN microscope operated at 120 kV. For sample preparation, 5 μL of each sample solution (typically at 0.5 mg/ml) was deposited on glow discharged continuous-film carbon-coated copper grids (Ted Pella Inc.), wicked off after 2 mins, and then stained with 5 μL Nano-W (methylamine tungstate, Nanoprobes Inc.) for 1 min before being wicked off. The grid was then left to dry before imaging. Measurements of nanofibrils lengths and widths were performed using DigitalMicrograph (Gatan Inc.).

Cryo-TEM imaging was performed on a JEOL 2100 FEG microscope operated at 200 kV. For sample preparation, 3 μL of each sample solution was deposited on a standard copper grid coated with a lacey carbon film and an ultrathin carbon film on top (Ted Pella Inc.), which was then plunge freezed using a Gatan Cryoplunge 3. Grids were stored in liquid nitrogen until being loaded into a pre-chilled cryogenic sample holder Gatan 626. The sample holder tip and grid were kept in liquid nitrogen throughout the transferring process and subsequent imaging. Minimum electron dose was used to avoid quick sample damage during the imaging. All images were recorded with a Gatan Ultrascan 2k × 2k CCD camera.

Fluorescence and polarization microscopy images were obtained on a Nikon TE2000-E inverted microscope. For fluorescence imaging, white light was coupled with DAPI and FITC filter cube sets. For birefringence imaging, two linear polarizers (one placed at the light source and one above the sample) were used to illuminate the samples with cross-polarized light.

### Nanomechanical characterization

Nanoindentaion measurements were performed on a Hysitron TriboIndenter with a nanoDMA transducer (Bruker). Samples were indented in load control mode with a peak force of 500 μN and a standard load-peak hold-unload function. Reduced modulus was calculated by fitting the unloading data (with upper and lower limits being 95 and 20%, respectively) using the Oliver-Pharr method and converted to Young’s modulus assuming a Poisson’s ratio of 0.33 for both silk and PS. For sample preparation, amorphous silk films were produced by drop casting 6 wt% silk fibroin suspension on 1 × 1 cm^2^ silicon wafers and dried naturally on the bench. Crystalline silk films were produced by soaking the silicon-supported amorphous silk films in 80% ethanol-water for 3 mins and then quickly dried under gentle air flow. PS films were fabricated by spin coating 20 wt% PS-xylene suspension on 1 × 1 cm^2^ silicon wafers at 3000 rpm for 1 mins, followed by heating at 100 ˚C for an hour to complete the solvent evaporation. Each type of sample was prepared and indented in triplets to ensure good fabrication repeatability. For each sample, indentation was performed at a total of 49 points (7 × 7 grid with an increment of 20 μm in both directions) to ensure statistical reliability of the modulus measurements.

AM-FM viscoelastic mapping was performed on a Cypher S AFM (Asylum Research) using AC160TS-R3 (Olympus) probes. Cantilever spring constant and sensitivity were calibrated using the built-in GetReal function of the Asylum Research software. A spin-coated PS film (roughness root mean square = 226 pm over an area of 5 × 5 μm^2^) on silicon was used as the reference sample (the Young’s modulus of which was 6.3 GPa based on the nanoindentation results) to calibrate the tip radius before each sample scan. Sample preparation, scan rate and image resolution were the same as that described for topography imaging in air. For each modulus map, histograms of the moduli at pixel positions corresponding to the peptide assemblies and silk nanofibrils were plotted and fitted with either a normal or log-normal distribution to determine the modulus average and standard deviation.

Nanomechanical measurements on the peptide assemblies and silk nanofibrils in water were performed by fast force mapping mode on a Cypher S AFM (Asylum Research) using BL-AC40TS (Olympus) probes. A 50 μL aliquot of diluted samples was dropped on freshly cleaved mica surface (*ϕ* = 10 mm, Ted Pella) and settled for 30 mins in a humid chamber before imaging. Cantilever spring constant and sensitivity in air were first calibrated using the built-in GetReal function of the Asylum Research software before immersing the cantilever in water, followed by a calculation of the cantilever sensitivity in water through capturing and fitting the thermal spectrum of the cantilever in water. All force maps were acquired at a scan rate of 0.5 Hz and a resolution of 256 × 256 pixels. Young’s modulus at each pixel was obtained by fitting the force-indentation curve with JKR model using Igor Pro (WaveMetrics Inc.). For each modulus map, histograms of the moduli at pixel positions corresponding to the peptide assemblies and silk nanofibrils were plotted and fitted with a log-normal distribution to determine the modulus average and standard deviation.

Nanoindentation of bulk silk nanofibrils gel in hydrated state was performed on a Cypher S AFM (Asylum Research) using a round-tip probe composed of a silicon nitride cantilever of 0.6 N/m nominal stiffness with a 0.6 μm diameter SiO_2_ bead attached at the end of the probe tip (Novascan Technologies). Before the experiment, cantilever sensitivity in air was calibrated by indenting a hard surface (a glass slide), followed by calibration of the spring constant through capturing and fitting the thermal spectrum of the cantilever in air. Then the cantilever was immersed in water and its sensitivity in water was again obtained through the thermal method. A 6 × 6 indentation grid over an area of 20 × 20 μm was performed at each location and a minimum of six different locations per sample were indented. Data acquisition was taken at a rate of 1.0 μm/s with a withdraw distance of 1.0 μm and a deflection trigger of 100 nm. Force-indentation curves were analyzed using Hertz, JKR, and Oliver-Pharr models, assuming a Poisson ratio of 0.33.

### Characterization of solution-state structures and properties

CD spectra were recorded from 185 to 260 nm using a JASCO J-1500 spectrometer, with each spectrum averaged from three consecutive scans. Samples of a concentration higher than 0.1 mg/ml were diluted to 0.1 mg/ml and measured in a 1 mm path length quartz cuvette (Starna Cells, Inc.). To estimate the fraction completion of templated assembly at any given time, the relevant CD spectrum was fitted as a linear combination of the initial spectrum at time 0 and the final spectrum at equilibrium time point where the CD spectrum remains constant thereafter. The fraction completion of templated assembly as a function of time was then fitted with an empirical logistic function commonly used for describing the kinetics of protein fibrils growth, from which the lag time and growth rate were extracted.

DLS measurements were performed on a DynaPro NanoStar Light Scatterer (Wyatt Technology). Samples of concentrations from 0.01 mg/ml to 1 mg/ml were measured in plastic cuvettes (UVette, Eppendorf). The laser was at 658 nm and its power was adjusted automatically for samples of different concentrations to an optimized range of counts by the built-in auto-attenuation capability. The acquisition time for each data point was 5 s, and 10 data points were acquired for each sample. The autocorrelation curve for each sample was examined to make sure there was no severe aggregates.

Fluorescence emission spectra were recorded using a FluoroLog-3 spectrofluorometer (Horiba Jobin Yvon). ANS emission spectra were collected from 420 to 600 nm with excitation at 350 nm, while for pyrene, excitation was set to 332 nm and emission was recorded from 350 to 420 nm. For all scans, both excitation and emission slit widths were 5 nm. Standard 10 mm path length spectrofluorometer cuvettes were used for loading the samples. The fluorophore concentrations used were 10 μm for ANS and 0.2 μm for pyrene, respectively. Fluorescence EEM were recorded with excitation from 250 to 500 nm and emission from 265 to 600 nm in 5 nm increments. For all EEM acquisition, sample concentration was 2 mg/ml and slit widths were set to 2 nm.

Viscosity measurements were performed on a rheometer (AR-G2, TA Instruments) at 22 ˚C with a cone plate of diameter 40 mm and cone angle 2˚. The evolution of viscosity was monitored at shear rates over a range of 0.1 to 3000 s^−1^ in which the residence time at each shear rate was sufficiently long to reach the final steady state flow. Oscillatory strain sweeps at a frequency of 1 Hz and with a strain range of 10^−4^–1 were performed to measure the storage and loss moduli in the linear viscoelastic region. A solvent trap cover was used to minimize water evaporation during the experiments.

### Characterization of materials crystallinity

ATR-FTIR was performed on a Nicolet 6700 FTIR spectrometer (Thermo Scientific) equipped with a liquid nitrogen-cooled microscope. Spectra were collected in reflection mode with ATR correction using a germanium crystal. All spectra were collected on dried samples from 4000 to 650 cm^−1^, with a resolution of 4 cm^−1^ and an accumulation of 64 scans. For the peptides used in this study, ATR-FTIR spectra were collected on both as-synthesized powders (lyophilized format) and dried films which were drop cast from a peptide-water suspension of ~ 8 mg/ml. Mapping was performed on a 1.2 × 1.2 cm^2^ area of the patterned silk film with an increment of 500 μm in both directions. The relative fractions of different secondary structures of silk fibroin were determined by Fourier self-deconvolution of the Amide I band (1705–1595 cm^−1^) and Gaussian curve-fitting of the deconvoluted spectra using Origin.

WAXS measurements were performed on a SAXSLAB instrument in transmission mode with a Dectris Pilatus3R 300 K detector set at a distance of 109.1 mm from the sample and a Rigaku 002 microfocus X-ray source producing Cu K_α1_ X-rays of wavelength 1.5409 Å. Samples were deposited on a thin mica window (5–7 μm thick, MOLMEX SCIENTIFIC INC) and dried before exposing to X-rays. The thin mica windows were chosen owing to its transparency to X-rays with no characteristic peaks in the scattering range of interest. Each spectrum was collected for 5 mins.

### Materials fabrication

For the fabrication of patterned silk films with predesigned crystallinity at selected areas, a PDMS sheet was first covered by a mask and plasma-treated to introduce polar functional groups (mainly SiOH) on the exposed areas, while keeping the surface property of the covered areas unchanged. Peptide suspensions of 3 mg/ml (or higher concentrations) were then pipetted onto the exposed areas following a predesigned pattern. After drying, the peptide assemblies were assumed to be immobilized on the PDMS surface through the formation of hydrogen bonds. Silk fibroin suspensions of 10 mg/ml (or higher concentrations) were then drop cast onto the peptide-modified PDMS sheet and incubated in a humid chamber for 24 hours, followed by natural drying on the bench to produce free-standing films or freeze-drying for SEM characterization.

Inkjet printing was performed on a piezoelectric inkjet printer (Dimatix DMP-2850, FUJIFILM) with cartridges of 10 pL nozzles (21 μm in diameter). Silk nanofibrils suspension was diluted to 50 μg/ml and filtered with cellulose acetate membranes of 0.45 μm pore size (VWR International) before loading into the cartridges. Custom designed jetting waveforms were used to optimize the jetting trajectory. Typically, drop spacing was set to 10 μm and three layers were printed to achieve uniform coverage of the printed pattern. Silicon wafers were used as the substrate.

Continuous printing of the silk gel was performed on a 3D bioprinter (INKREDIBLE, CELLINK). Typically, straight needles with an inner diameter of 0.3 mm (25-gauge, McMaster Carr) and a printing speed of 5 mm/s were used. Extrusion pressures in the range of 10–50 kPa were applied and adjusted for silk gels of different concentrations. Standard glass slides were used as the substrate.

## Supplementary information

Supplementary Information

## Data Availability

The data that support the findings of this study are available from the corresponding author upon reasonable request.
