# Supplementary material for: Polypeptide templating for designer hierarchical materials
Source: Nat Commun. 2020 Jan 17;11:351. doi: 10.1038/s41467-019-14257-0 (PMC6969164; doi:10.1038/s41467-019-14257-0)
Supplement: Supplementary file 1 — Supplementary Information [file 41467_2019_14257_MOESM1_ESM.pdf]

Supplementary Information

Polypeptide templating for designer hierarchical materials

Sun et al.

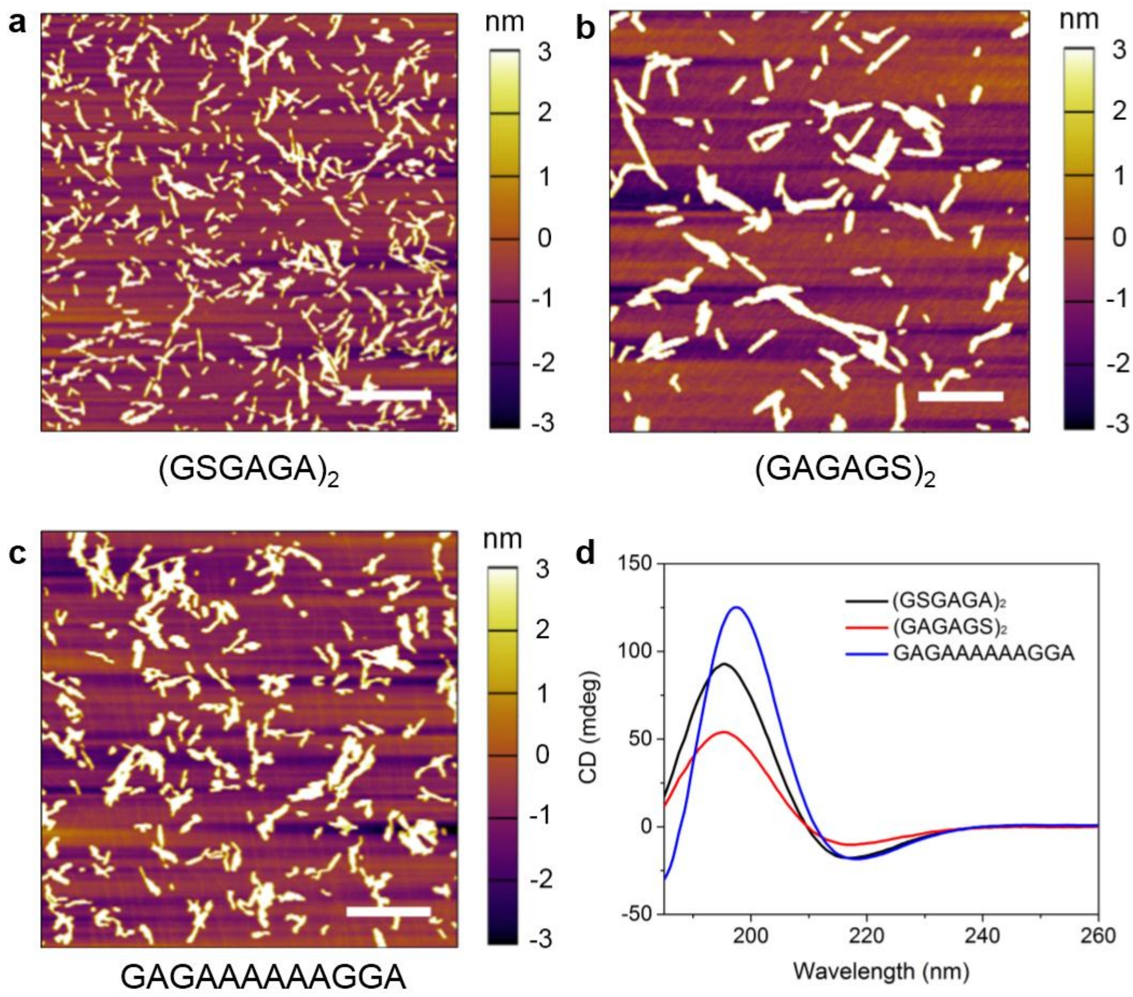

Supplementary Figure 1. Supramolecular assembly of dodecapeptides $(GSGAGA)_2$, $(GAGAGS)_2$ and GAGAAAAAAGGA. Representative atomic force micrographs of **a,** $(GSGAGA)_2$ self-assembled in water, **b,** $(GAGAGS)_2$ self-assembled in 1mM NaOH, and **c,** GAGAAAAAAGGA self-assembled in water, showing formation of relatively irregular supramolecular oligomers. Scale bars, 400 nm. **d,** CD spectra of $(GSGAGA)_2$, $(GAGAGS)_2$ and GAGAAAAAAGGA at 0.1 mg/ml, depicting similar β-pleated sheet structures for the three peptide assemblies.

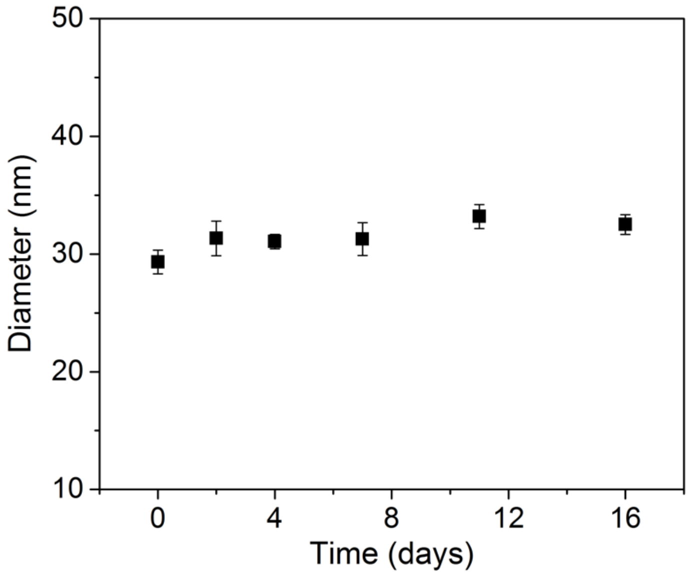

Supplementary Figure 2. Effective hydrodynamic diameters of $(GAGSGA)_2$ nanowhiskers measured by DLS, showing that the nanowhiskers are stable over time. Error bars represent standard deviation.

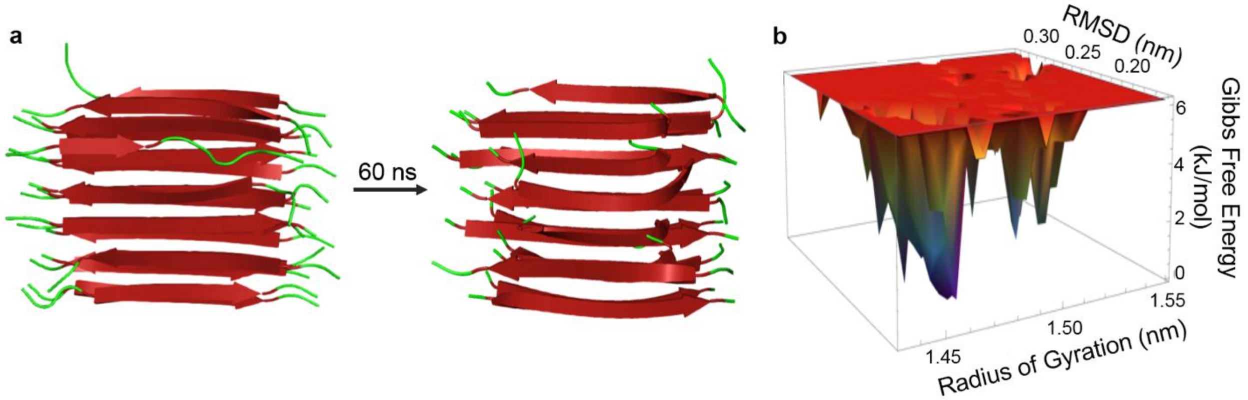

Supplementary Figure 3. Molecular dynamics (MD) simulation of the structural evolution of a representative $(GAGSGA)_2$ oligomer composed of eighteen peptides. a, Initial and equilibrated structures of the simulated $(GAGSGA)_2$ oligomer. **b,** Free energy landscape of the system dynamics, indicating that energy was minimized in a β-sheet configuration.

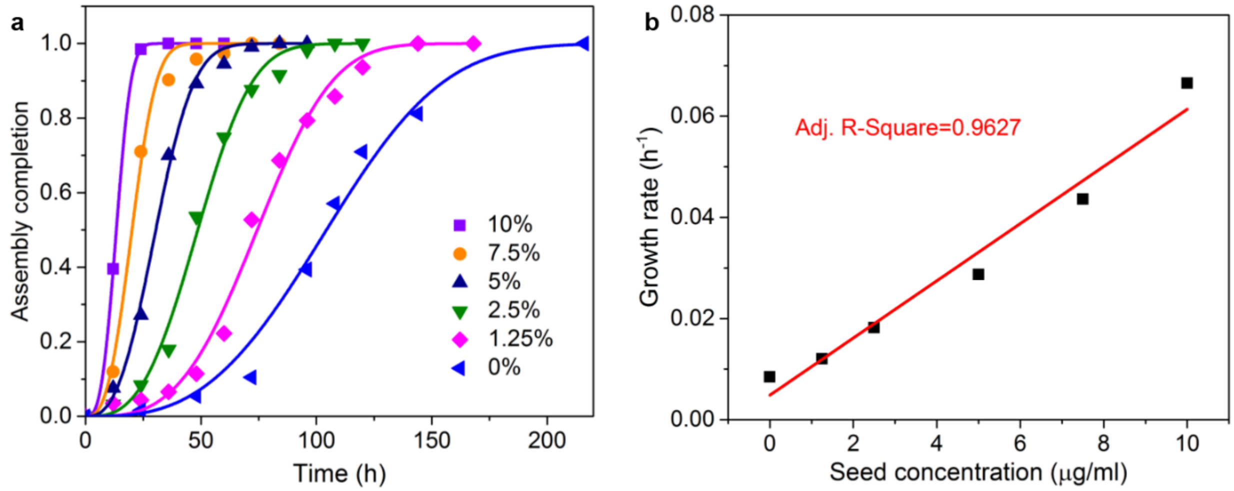

Supplementary Figure 4. Evaluation of the kinetics of templated crystallization with JMAK model. **a,** The same kinetics profiles of templated crystallization of silk fibroin on $(GAGSGA)_2$ nanowhiskers, fitted with a modified JMAK model $y = 1 - \exp\left(-(Kt)^{d+1}\right)$. **b,** Fibrils growth rate K extracted from the JMAK model fitting and plotted against $(GAGSGA)_2$ concentration.

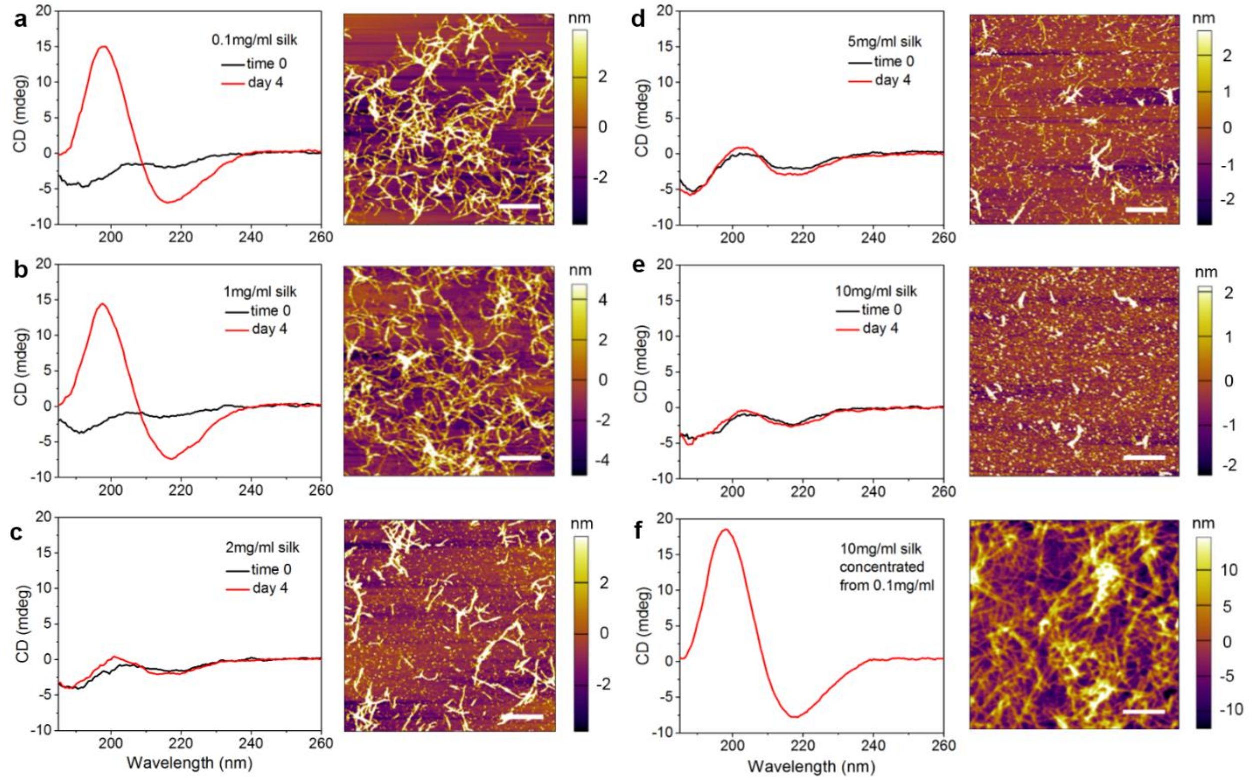

Supplementary Figure 5. Effect of silk fibroin (i.e. monomer) concentration on the kinetics of templated crystallization. CD spectra and corresponding atomic force micrographs of silk fibroin at increasing concentrations seeded by $(GAGSGA)_2$ nanowhiskers at 5%. The investigated silk fibroin (i.e. monomer) concentrations were 0.1, 1, 2, 5 and 10 mg/ml, as shown in panels **a-e**, and the AFM images were taken at day 4. A random coil to β-sheet transition of silk fibroin occurred at 0.1 and 1 mg/ml, but not at 2, 5 and 10 mg/ml within the same time frame. The degree of nanofibrillization also decreases with increasing silk fibroin concentrations, indicating that templated crystallization becomes less efficient at higher silk fibroin concentrations. **f**, CD spectrum and corresponding AFM image of silk fibroin at 10 mg/ml concentrated from 1 mg/ml through a simple water evaporation process. Scale bars, **a-e**, 1 μm. **f,** 400 nm.

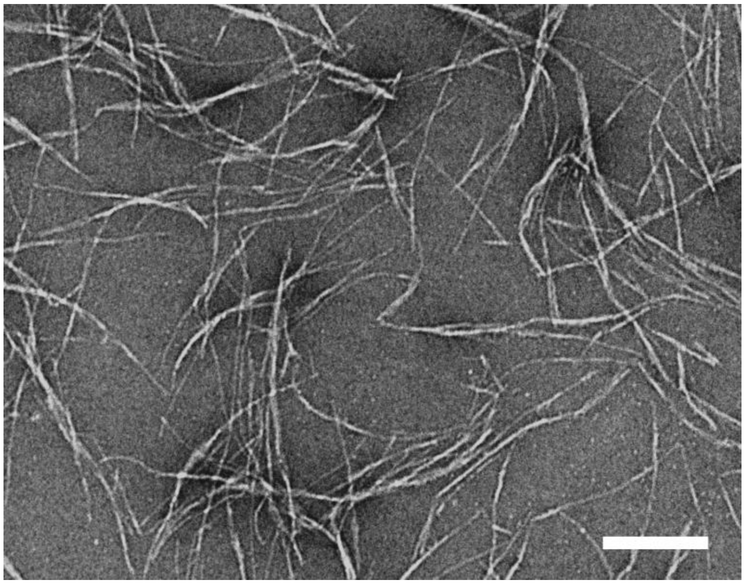

Supplementary Figure 6. Negative-stain TEM image of silk nanofibrils formed by natural aging (i.e., in the absence of templates) after 1 month, for comparison with the $(GAGSGA)_2$-templated silk nanofibrils shown in Fig. 3g. Scale bar, 200 nm.

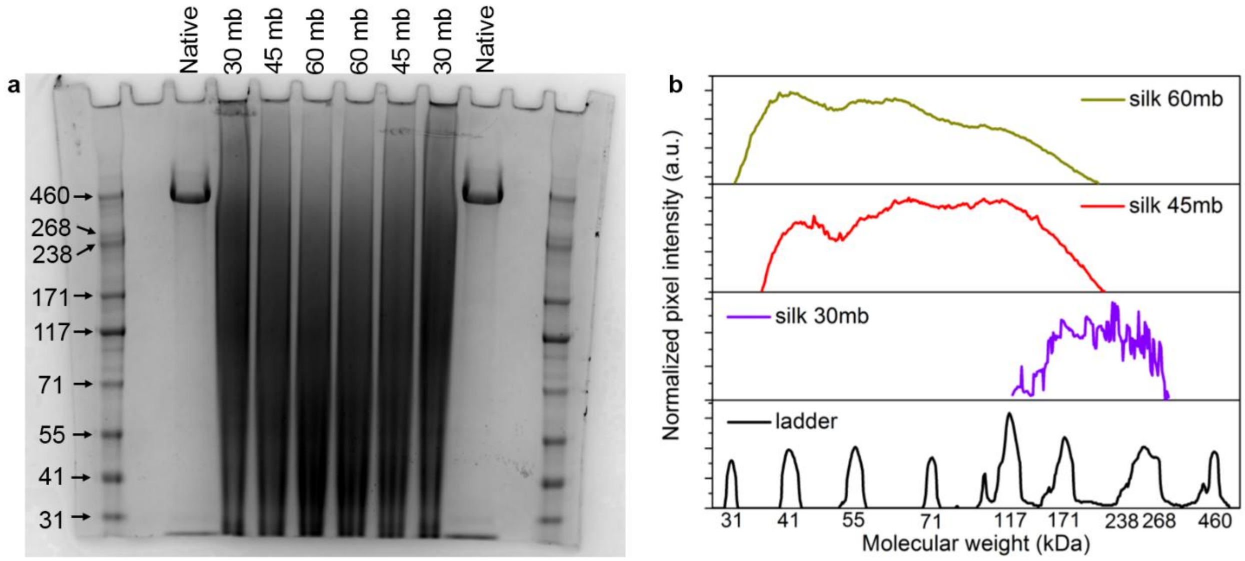

Supplementary Figure 7. Molecular weight distributions of native and regenerated silk fibroin measured by SDS-PAGE. a, Lane 1 and 12: HiMark™ Pre-stained Protein Standard; Lane 2 and 11: empty; Lane 3 and 10: Native silk fibroin extracted from the gland of *Bombyx mori* silkworms; Lane 4 and 9: Regenerated silk fibroin degummed for 30 minutes; Lane 5 and 8: Regenerated silk fibroin degummed for 45 minutes; Lane 6 and 7: Regenerated silk fibroin degummed for 60 minutes. **b,** Quantitative molecular weight distribution plot obtained by pixel intensity analysis of the gel image.

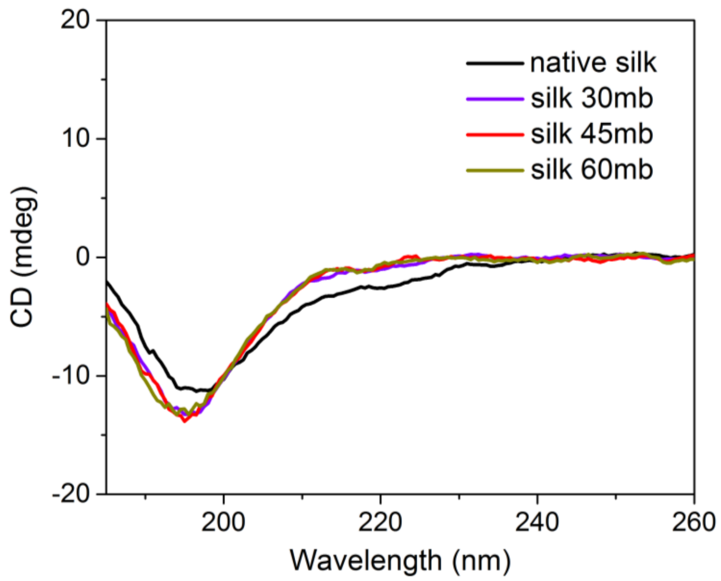

Supplementary Figure 8. Structural characterization of native and regenerated silk fibroin by CD, showing similar random coils dominant conformation regardless of the protein extraction time.

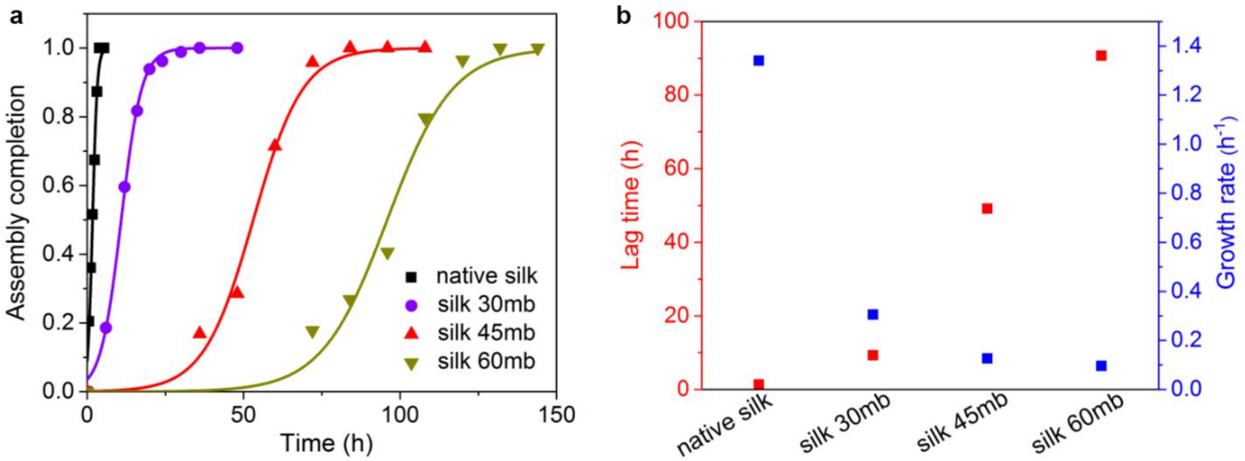

Supplementary Figure 9. Effect of silk fibroin molecular weight (MW) on the kinetics of templated crystallization. a, Fraction completion of templated assembly as a function of time for silk fibroin of different MW. Data points were calculated from time-series CD spectra and fitted with a logistic function (solid lines). **b,** Lag time and growth rate extracted from the kinetics model fitting and plotted against silk fibroin of different MW.

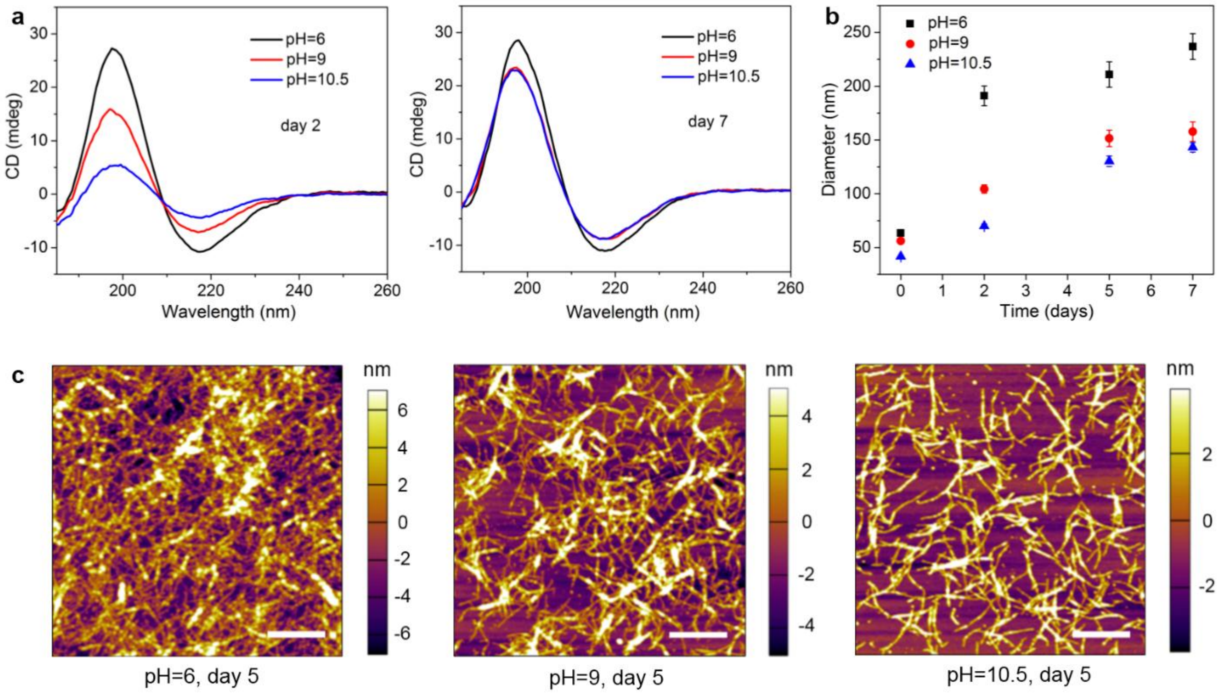

Supplementary Figure 10. Effect of milieu pH on the kinetics of templated crystallization. a, CD spectra of silk fibroin seeded by 10% $(GAGSGA)_2$ nanowhiskers at different pH collected on day 2 (left) and day 7 (right), showing a slower templated crystallization at increased pH. **b,** Effective hydrodynamic diameters of silk fibroin seeded by $(GAGSGA)_2$ nanowhiskers at different pH over time. Error bars represent standard deviation. **c,** Representative atomic force micrographs of the assembled silk nanofibrils at different pH on day 5, showing that increased milieu pH restricts nanofibrils growth in length and mitigates the overall aggregation and association of the silk nanofibrils. Scale bars, 1 μm.

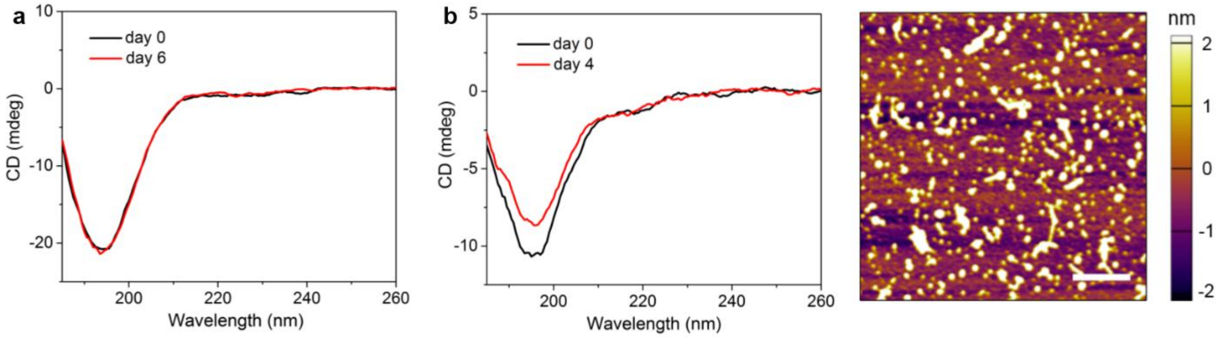

Supplementary Figure 11. Heat-induced disassembly of $(GAGSGA)_2$ nanowhiskers and the associated templating effects. a, CD spectra of 0.1 mg/ml $(GAGSGA)_2$ solution after being heated at 80 ^{o}C for 20 minutes, showing that β-sheet nanowhiskers were disrupted and the disassembled peptides didn't reassemble for at least 6 days. **b,** CD spectra of silk fibroin seeded by 5% disassembled $(GAGSGA)_2$ (left), showing no random coil to β-sheet transition up to day 4. Corresponding atomic force micrograph on day 4 (right), depicting no nanofibrils formation. Scale bar, 400 nm.

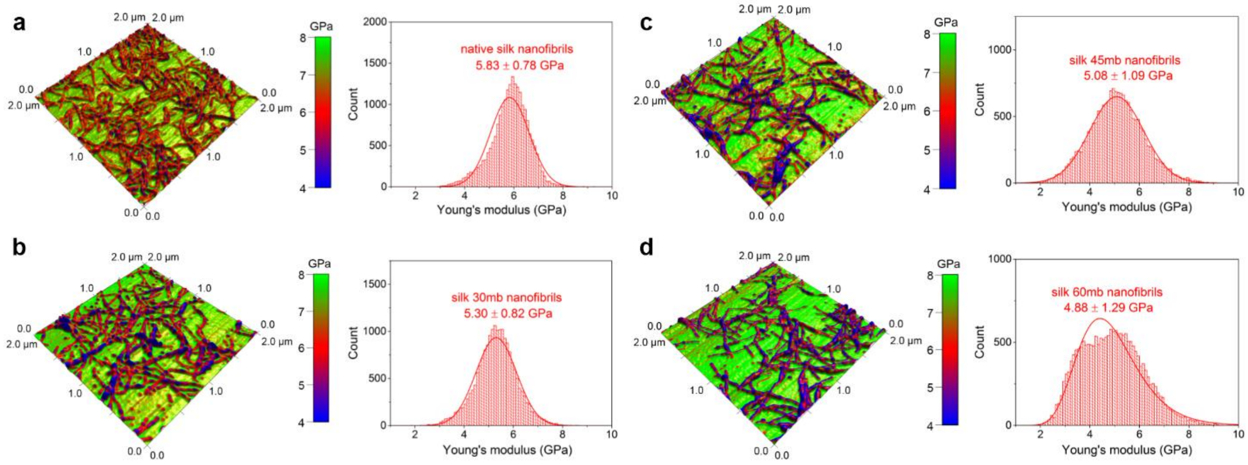

Supplementary Figure 12. Effect of silk fibroin molecular weight on the nanomechanical properties of the assembled fibrils. Young's modulus map overlaid on 3D topography (left) and histograms of the Young's moduli (right) for nanofibrils assembled from **a,** native silk fibroin; **b,** regenerated silk fibroin degummed for 30 minutes (silk 30mb); **c,** regenerated silk fibroin degummed for 45 minutes (silk 45mb) and **d,** regenerated silk fibroin degummed for 60 minutes (silk 60mb).

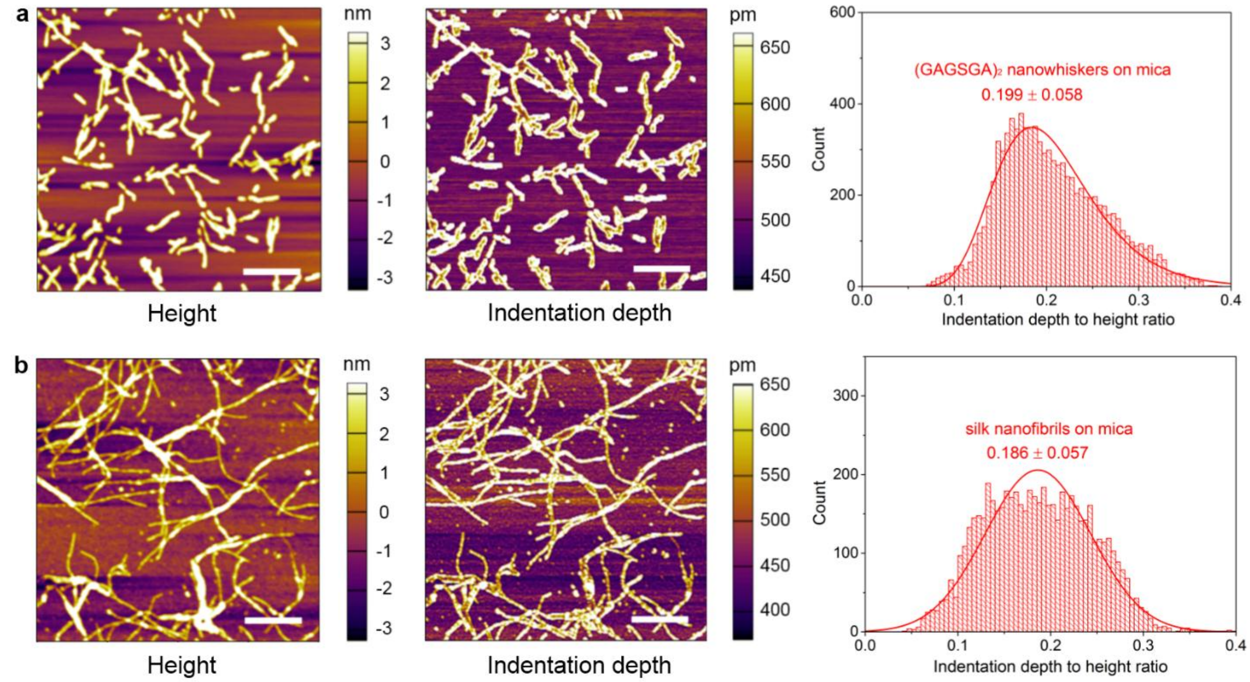

Supplementary Figure 13. Maps of height (left) and indentation depth (middle), and histograms of indentation depth to height ratio (right) for **a,** $(GAGSGA)_2$ nanowhiskers deposited on mica and **b,** silk nanofibrils (templated by $(GAGSGA)_2$ nanowhiskers) deposited on mica. Scale bars, 400 nm.

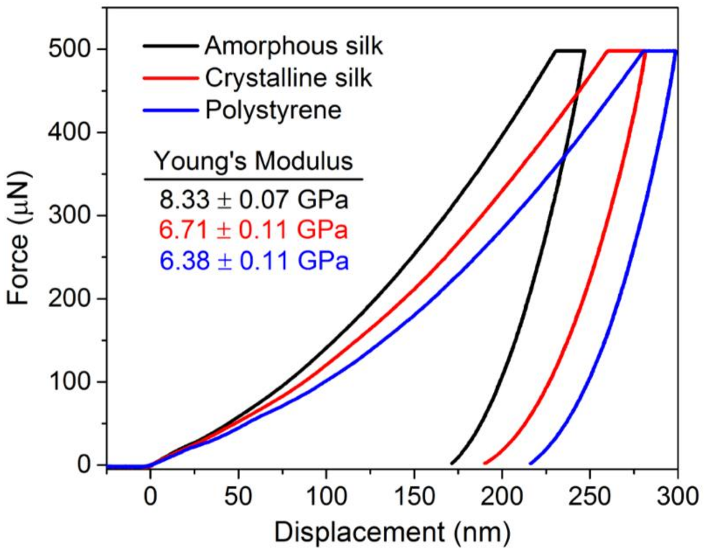

Supplementary Figure 14. Force-displacement curves of drop-cast amorphous silk films, crystalline silk films obtained by 80% ethanol-water treatment and spin-coated polystyrene (PS) films, measured by nanoindentation.

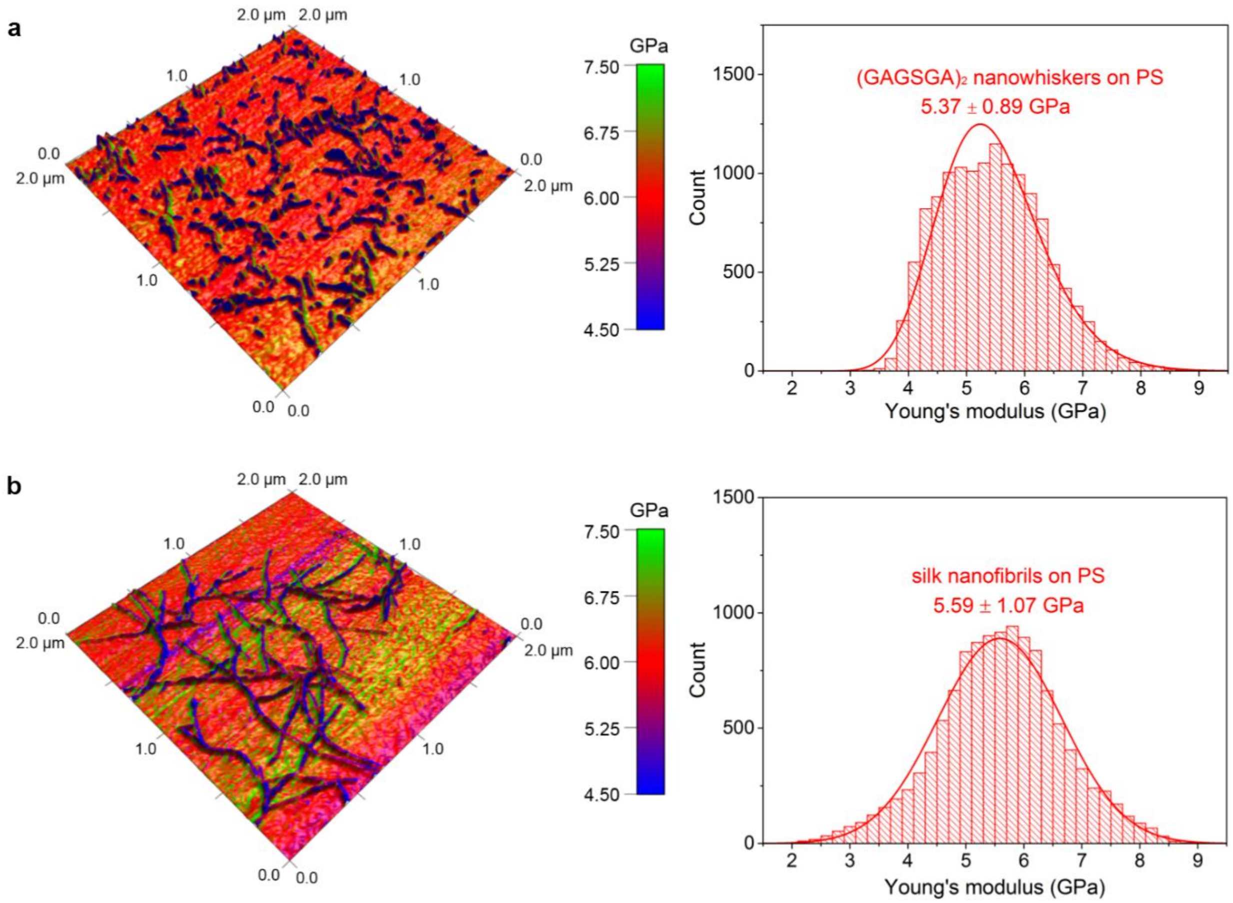

Supplementary Figure 15. Young's modulus map overlaid on 3D topography obtained by AM-FM (left) and histograms of the Young's moduli (right) for **a,** $(GAGSGA)_2$ nanowhiskers deposited on PS; **b,** silk nanofibrils (templated by $(GAGSGA)_2$ nanowhiskers) deposited on PS.

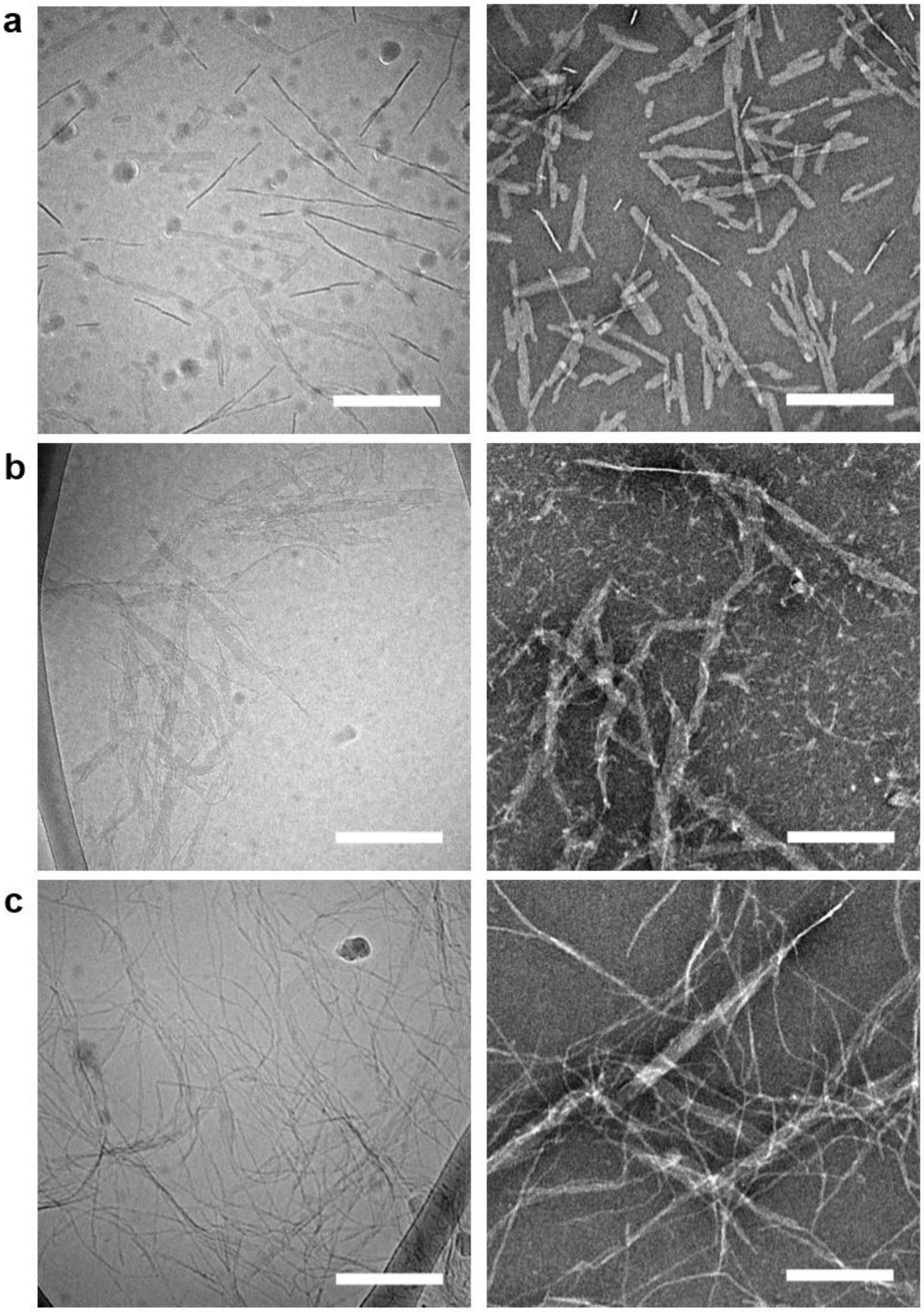

Supplementary Figure 16. Effect of drying on the assembly and morphology of peptide/silk fibroin assemblies. Cryo-EM image (left) and negative-stain TEM image (right) of the same sample at the same magnification for: **a, (**GAGSGA)$_2$ nanowhiskers. **b, (**GAGSGA)$_2$-templated silk nanofibrils at 1h after mixing. **c, (**GAGSGA)$_2$-templated silk nanofibrils at 48h after mixing. No apparent difference in the morphology and dimensions of the (GAGSGA)$_2$ nanowhiskers and the tempalted silk nanofibrils between hydrated and dried state was observed, except that more (GAGSGA)$_2$ nanowhiskers were captured in the upright position (shown by the darker and thinner lines in the upper-left cryo-EM image) during the freezing process. Scale bars, 200 nm.

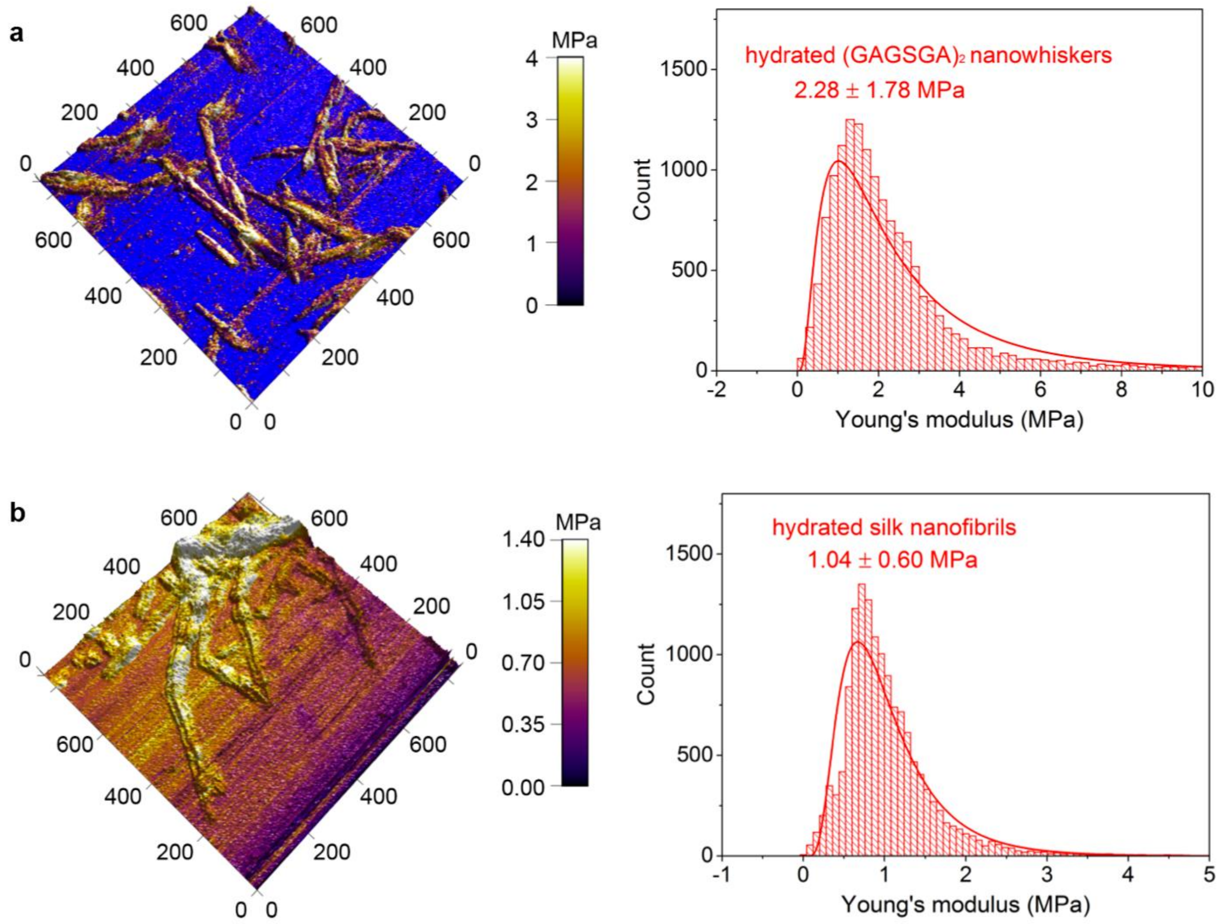

Supplementary Figure 17. Nanomechanical characterization of hydrated peptide/silk fibroin assemblies by AFM. Young's modulus maps acquired with fast force mapping mode and calculated by analyzing the force-indentation curves at each pixel with JKR model, overlaid on 3D topography (left); and histograms of the Young's moduli (right) for **a,** $(GAGSGA)_2$ nanowhiskers in water. **b,** $(GAGSGA)_2$-templated silk nanofibrils in water.

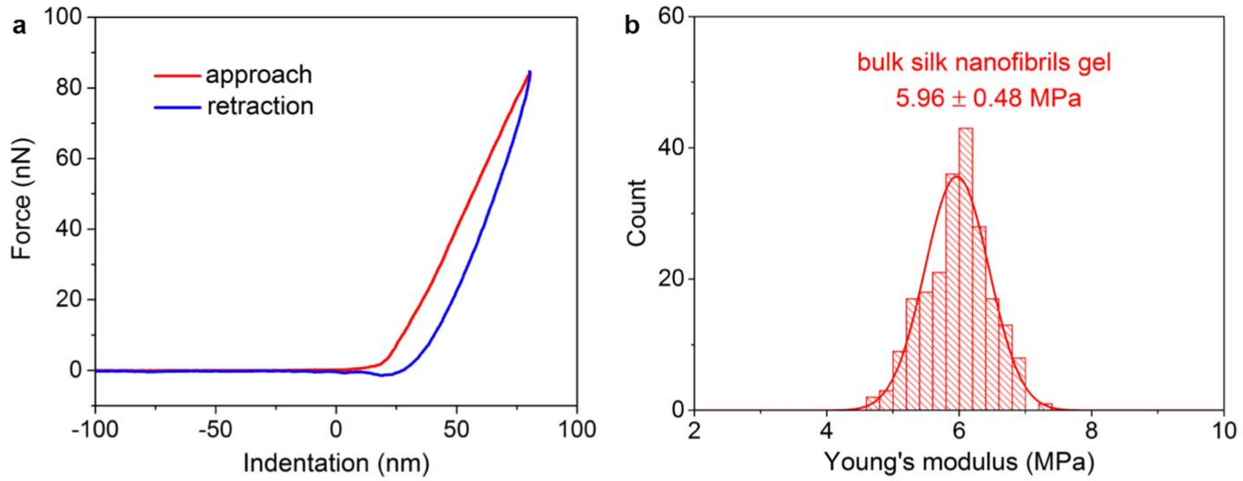

Supplementary Figure 18. Nanoindentation on hydrated silk nanofibrils gel with AFM. a, Representative force-indentation curve on hydrated gels formed by templated assembly of silk fibroin on $(GAGSGA)_2$. **b,** Histograms of Young's moduli obtained by fitting the force-indentation curves with Hertz model.

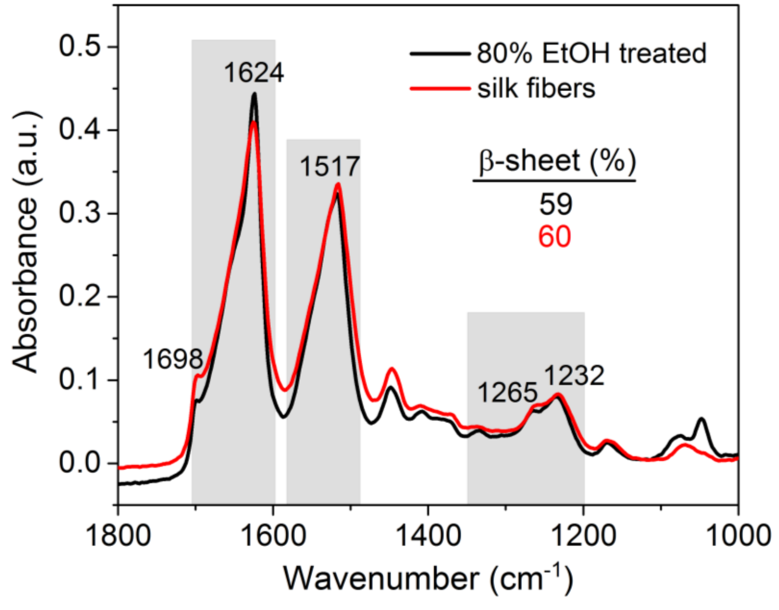

Supplementary Figure 19. ATR-FTIR spectra of drop cast silk films treated by 80% EtOH for 30 minutes and silk fibers degummed from *Bombyx mori* cocoons for 30 minutes, respectively. The beta-sheet content of each spectrum was calculated and compared to that of silk fibroin templated by $(GAGSGA)_2$ and HBSP seeds (Fig. 6b).

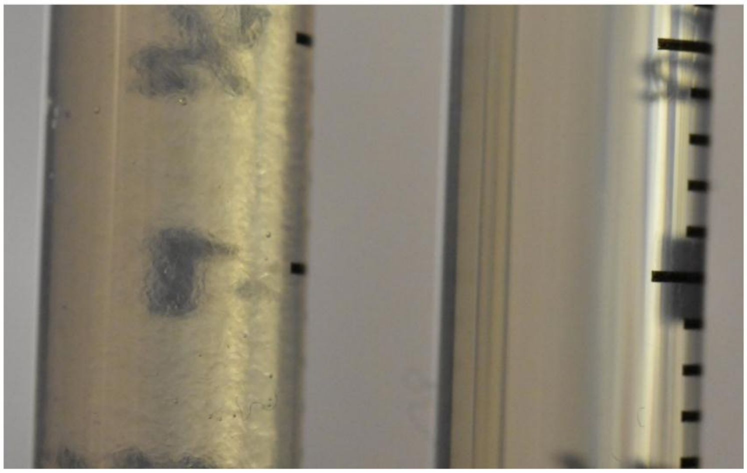

Supplementary Figure 20. Image of the concentrated silk gel for bioprinting (left) and regenerated silk fibroin suspension of the same concentration (right, for comparison) loaded in standard 3mL syringes.

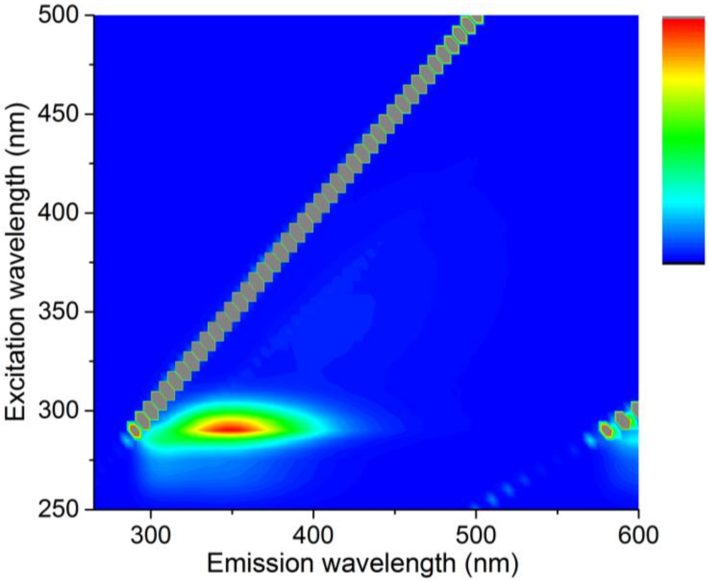

Supplementary Figure 21. Fluorescence excitation-emission matrix (EEM) of regenerated silk fibroin solution at 2 mg/ml, showing excitation and emission maxima at 290 and 350 nm respectively, due to the presence of tyrosine and tryptophan in silk fibroin. The color scale represents fluorescence intensity (arbitrary unit).

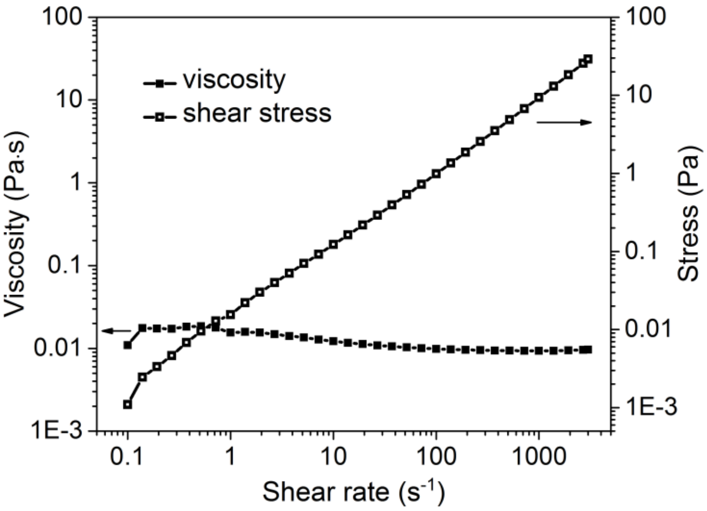

Supplementary Figure 22. Shear viscosity and stress of 7 wt% regenerated silk fibroin solution as a function of shear rate, depicting little shear-thinning behavior. Storage and loss moduli measurement is meaningless in this case, with negative storage modulus reported due to minimal elasticity of the regenerated silk fibroin solution.

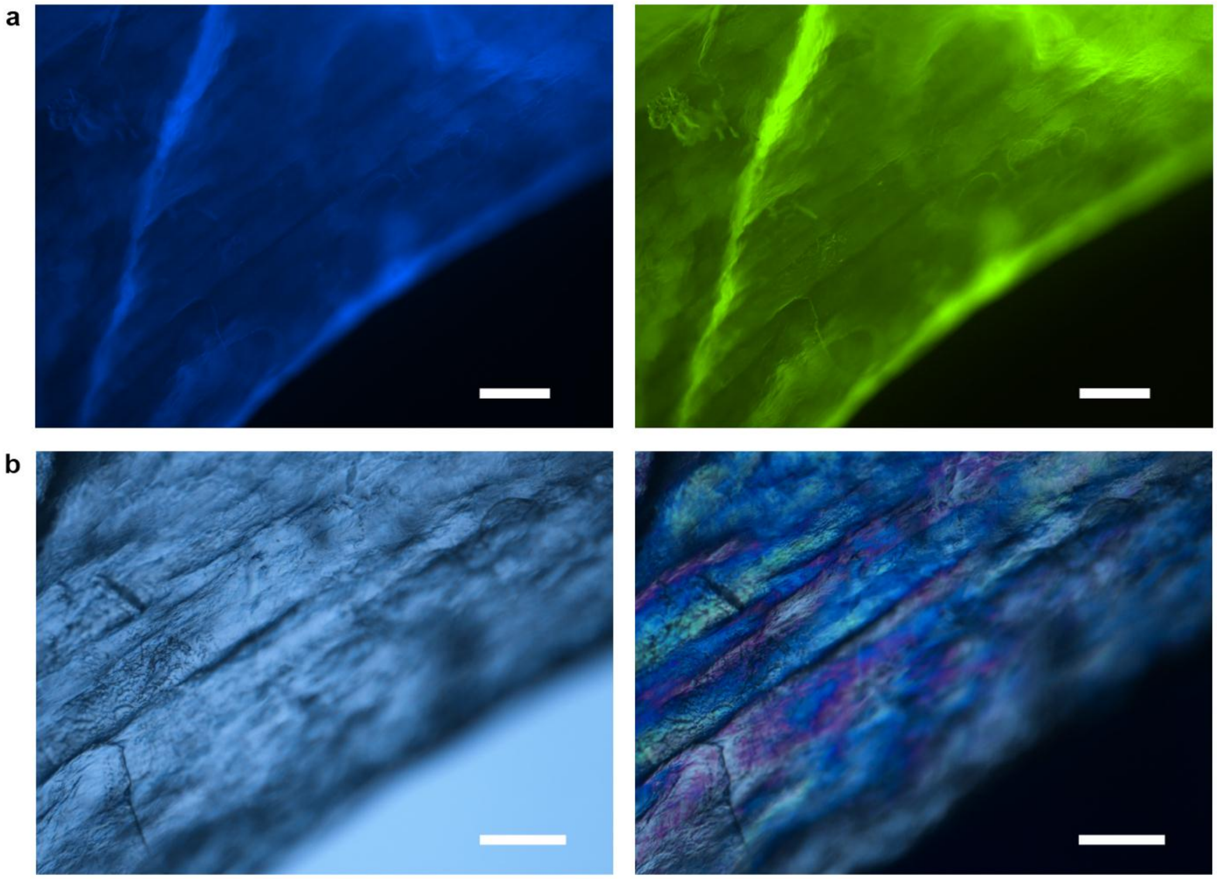

Supplementary Figure 23. Microscopic characterization of printed silk materials. **a,** Fluorescence microscopy images captured with different filters (left, DAPI; right, FTIC), showing enhanced intrinsic fluorescence of the printed silk construct. Scale bars, 200 μm. **b,** Bright-field (left) and polarization microscopy image (right), depicting birefringence of the same silk construct. Scale bars, 100 μm.

Supplementary Table 1. Summary of the molecular weight (MW) distribution parameters calculated from pixel intensity analysis of the SDS-PAGE gel image.

	MW range (kDa)	weight average MW (kDa)	number average MW (kDa)	polydispersity index (PDI)
silk 30mb	310 - 120	210	198	1.06
silk 45mb	210 - 37	118	93	1.27
silk 60mb	200 - 31	111	85	1.31
